# Integrative Multi-Database Bioinformatics Characterization of *FANCE* in Hepatocellular Carcinoma: Prognostic, Epigenetic, Immune, and ncRNA Associations

**DOI:** 10.3390/medicina62101988

**Published:** 2026-10-14

**Authors:** Yeong Chae Kim, Kyu-Shik Lee, Hye-Ran Kim, Jongwan Kim

**Affiliations:** 1 Department of Anatomy, College of Medicine, Dongguk University, Gyeongju 38066, Republic of Korea; rladudco0816@dumc.or.kr; 2 Department of Pharmacology, College of Medicine, Dongguk University, Gyeongju 38066, Republic of Korea; there1@dongguk.ac.kr; 3 Department of Biomedical Laboratory Science, Dong-Eui Institute of Technology, Busan 47230, Republic of Korea

**Keywords:** liver hepatocellular carcinoma, Fanconi anemia complementation group E, Fanconi anemia, DNA methylation, ncRNA networks, immune infiltration, anticancer-drug response

## Abstract

*Background and Objectives:* The Fanconi anemia complementation group E (FANCE) is a protein component of the Fanconi anemia (FA) DNA repair complex and contributes to genome integrity. However, its molecular and clinical relevance in liver hepatocellular carcinoma (LIHC) remains incompletely understood. This study characterized the *FANCE* expression, prognosis, epigenetics, immune responses, noncoding RNA (ncRNA) networks, and other related molecular processes in LIHC. *Materials and Methods:* An integrative multi-database bioinformatics analysis was conducted using public databases and web-based platforms. FANCE expression, DNA methylation, co-expression profiles, and functional enrichment; immune cell infiltration; miRNA–ncRNA, chemical–gene, and protein–protein interactions; treatment response; and survival were evaluated. *Results: FANCE* expression was higher in LIHC than in non-tumor liver tissues, and this was linked to shorter survival duration. Promoter methylation was lower in LIHC, and select CpG sites were related to *FANCE* expression and survival. Immune analyses identified heterogeneous relationships between FANCE and inferred immune cell populations. hsa-miR-101-3p was reduced in LIHC, negatively correlated with FANCE, and linked to prolonged survival; subsequent network analysis identified candidate lncRNAs, small ncRNAs, circRNA-linked genes, and pseudogenes. FANCE-related transcriptional profiles were enriched in the cell cycle, DNA replication, FA, and other DNA-repair processes, whereas negatively correlated genes were mainly linked to hepatic metabolic functions. Exploratory treatments identified drug-dependent survival patterns related to FANCE expression and elevated levels in responders to specific immune-checkpoint inhibitors, although the discriminatory performance was modest. *Conclusions:* Increased *FANCE* expression in LIHC was linked to molecular features involving DNA methylation, immune cell composition, ncRNA-related interactions, and DNA repair programs as well as shorter survival. These findings provide an integrated molecular context for *FANCE* in LIHC but do not establish a causal regulation or therapeutic predictive value. Independent clinical and experimental validation studies are warranted to determine its biological and clinical significance.

## 1. Introduction

Liver hepatocellular carcinoma (LIHC), the predominant histological subtype of primary liver cancer, remains a major cause of cancer-related mortality worldwide [1]. There have been several advances in surveillance, surgical resection, liver transplantation, locoregional treatment, molecularly targeted therapy, and immunotherapy for LIHC; however, long-term clinical outcomes remain unsatisfactory because of late diagnosis, frequent recurrence, underlying hepatic dysfunction, treatment resistance, and substantial inter- and intratumoral heterogeneity [2,3]. LIHC is frequently associated with chronic liver disease and cirrhosis and exhibits substantial clinical and biological heterogeneity; this considerably challenges the prediction of pathogenesis and therapeutic outcomes [4,5]. At the molecular level, LIHC is characterized by diverse genomic, epigenetic, and transcriptomic alterations along with remodeling of the tumor microenvironment, all of which contribute to tumor development, progression, and therapeutic response [6,7,8]. Characterizing the molecular alterations associated with aggressive tumor behavior may therefore help improve prognostic assessment and therapeutic selection for LIHC. Maintenance of genomic integrity is particularly crucial in hepatocarcinogenesis as chronic inflammation, oxidative stress, replication stress, and accumulated DNA damage can promote genomic instability and tumor evolution [9,10,11,12,13,14]. The Fanconi anemia (FA) DNA repair pathway is a highly conserved DNA-damage response pathway that plays a critical role in the recognition and repair of interstrand cross-links and replication-associated lesions [15,16,17]. The FA complementation group E (FANCE) is an essential component of the FA core complex that contributes to the complex’s assembly and facilitates the recruitment and monoubiquitination of the downstream *FANCD2* [18,19,20]. Through these functions, FANCE participates in coordinating DNA damage recognition, replication-associated repair, and chromosome stability maintenance. Therefore, FANCE dysregulation may influence cell-level responses to genomic stress and potentially contribute to malignant progression.

The clinical relevance of FANCE in cancer has begun to receive attention. A previous study reported increased *FANCE* expression in LIHC, an association between elevated expression and unfavorable patient survival, and an enrichment of proliferation-related transcriptional programs [21]. Pan-cancer analysis also suggested *FANCE* expression to be associated with prognostic and immune-related characteristics in various malignancies [22]. These observations support the potential role of FANCE in cancer biology and establish its expression and prognostic relevance in LIHC. However, a broader molecular context linked to *FANCE* dysregulation during LIHC remains insufficiently characterized. In particular, the association of FANCE with epigenetic regulation, non-coding RNA (ncRNA)-associated regulatory networks, tumor immune microenvironment, and coordinated molecular pathways has not been comprehensively characterized by multiple complementary public databases.

Epigenetic and post-transcriptional processes are important regulatory mechanisms in LIHC [23,24]. Aberrant DNA methylation can modify gene expression and contribute to distinct molecular and clinical tumor phenotypes; moreover, the potential of specific methylation patterns as diagnostic and prognostic biomarkers has been examined [23,25]. In addition, ncRNAs act as another major layer of gene regulation. MicroRNAs (miRNAs) regulate target transcripts at the post-transcriptional level, whereas long non-coding RNAs (lncRNAs), circular RNAs (circRNAs), pseudogene-derived transcripts, and other ncRNAs can participate in interconnected regulatory networks involving miRNAs and protein-coding genes [26,27,28,29,30]. Dysregulation of these networks has been associated with proliferation, apoptosis, invasion, metastasis, and therapeutic resistance in LIHC. Therefore, an integrated evaluation of DNA methylation and ncRNA-associated interactions can provide further insight into the regulation of altered *FANCE* expression.

The tumor immune microenvironment substantially contributes to LIHC progression and therapeutic responsiveness, with differences in the abundance and functional state of tumor-infiltrating immune cells (TICs) found to be associated with disease progression, survival, and responses to immunotherapy [31,32]. Accordingly, evaluating the relationship between *FANCE* expression and immune cell infiltration may help determine whether altered FANCE expression is associated with immune characteristics unique to LIHC. Moreover, DNA-damage response pathways are closely associated with cell proliferation and therapeutic response; therefore, characterizing FANCE-associated co-expression patterns, molecular functions, interaction networks, and drug-related features may further increase our understanding of its biological and potential therapeutic relevance.

The present study aimed to comprehensively characterize *FANCE* and its associated molecular features in LIHC using multiple public databases. *FANCE* mRNA and protein expression, clinicopathological characteristics, and prognostic significance were evaluated, followed by DNA methylation and immune cell infiltration analyses. *FANCE*-associated miRNAs and their related ncRNA-based networks including lncRNAs, circRNAs, pseudogenes, and other non-coding transcripts were also examined to characterize any potential post-transcriptional regulatory associations. Co-expression, functional enrichment, prognostic gene set, and protein–protein interactions were analyzed to characterize the molecular pathways and interaction networks associated with FANCE. Finally, drug responses and chemical interactions were examined to assess potential therapeutic associations. Through these complementary multi-database analyses, we aimed to define the broader molecular and clinical context of *FANCE* in LIHC.

## 2. Materials and Methods

### 2.1. FANCE mRNA Expression in LIHC

Tumor Immune Estimation Resource 3.0 (TIMER3; https://compbio.cn/timer3/; accessed on 23 May 2026) was used to compare the *FANCE* mRNA levels in tumor and adjacent normal tissues in The Cancer Genome Atlas (TCGA) cancer types [33]. In the TIMER3 Gene_DE module, differential expression between TCGA tumor and adjacent normal tissues was calculated using edgeR based on RNA-seq raw counts, with expression levels displayed as log2(transcripts per million [TPM] + 1). The expression in LIHC and normal liver tissues was further examined using OncoDB 2.0 (https://oncodb.org/; accessed on 13 May 2026), which incorporates TCGA and Genotype-Tissue Expression (GTEx) data [34]. For RNA expression analyses, OncoDB processes TCGA and GTEx RNA-seq data using a common bioinformatics pipeline and normalizes gene-level expression as TPM, thereby providing consistently processed expression data for tumor–normal comparisons. UALCAN (https://ualcan.path.uab.edu/; accessed on 13 May 2026) was used to conduct additional comparisons within the TCGA-LIHC cohort according to sample type and available clinicopathological variables, including age, sex, race, body weight, tumor grade, disease stage, nodal metastasis status, histological subtype, and TP53 mutation status [35,36]. Statistical comparisons between groups were performed using the unpaired two-sample *t*-test implemented in UALCAN.

### 2.2. FANCE Protein Expression in LIHC

FANCE protein expression was examined based on immunohistochemical data available from the Human Protein Atlas (HPA; https://www.proteinatlas.org/; version 25.1; accessed on 6 July 2026) [37]. Representative images from three normal liver and six LIHC specimens stained with the FANCE antibody CAB014893 were obtained from the HPA. The HPA-reported staining intensity, quantity, and subcellular localization were recorded for each specimen.

### 2.3. Prognostic Analysis of FANCE in LIHC

The relationship between *FANCE* expression and patient survival was examined using the Kaplan–Meier (KM) Plotter (https://kmplot.com/analysis/index.php?p=service&cancer=liver_rnaseq; accessed on 13 May 2026) [38]. Overall survival (OS), progression-free survival (PFS), recurrence-free survival (RFS), and disease-specific survival (DSS) of the available clinicopathological subgroups were assessed. Patients were divided into high- and low-FANCE expression groups using the “auto select best cutoff” option implemented in KM Plotter, with the cutoff determined separately for each survival analysis. Survival differences between the high- and low-FANCE expression groups were evaluated using the log-rank test implemented in the platform, and hazard ratios (HRs) with 95% confidence intervals (CIs) were obtained. The effective sample sizes were 364 for OS, 370 for PFS, 316 for RFS, and 362 for DSS. Subgroup-specific sample sizes were provided by KM Plotter according to the selected clinicopathological parameters. The subgroup analyses were based on nominal *p*-values without correction for multiple hypothesis testing and were therefore considered exploratory. Using Gene Set Cancer Analysis (GSCA; https://guolab.wchscu.cn/GSCA/#/; accessed on 13 May 2026), complementary survival information, including disease-free interval (DFI), was determined [39]. For GSCA survival analysis, patients were divided into high- and low-FANCE expression groups according to the median expression level. Survival differences were evaluated using the log-rank test and Cox proportional hazards regression implemented in GSCA. The effective sample sizes were 370 for OS and PFS, 277 for DFI, and 319 for DSS. Multivariable Cox proportional hazards analysis was additionally performed using the Gene_Outcome module of TIMER3 to examine the association between *FANCE* expression and OS after adjustment for the available clinical covariates of age, gender, and tumor stage. The analysis included 365 patients with complete data and 130 death events. Adjusted HRs with 95% CIs and *p*-values were obtained from the Cox model. To examine whether the association between *FANCE* expression and OS was distinguishable from a general proliferation-related signal, an additional multivariable Cox analysis was performed using the Gene Expression Profiling Integrative Analysis 3 (GEPIA3; https://gepia3.bioinfoliu.com/survanl/multi/; accessed on 30 September 2026) with *FANCE* and *MKI67* expression included simultaneously in the model. The prognostic relevance of *FANCE* across additional cancer cohorts was examined using PrognoScan (http://dna00.bio.kyutech.ac.jp/PrognoScan/; accessed on 13 May 2026), and datasets with significant associations between *FANCE* expression and survival were recorded [40]. A total of 89 survival-analysis results from 59 datasets were examined. Results with a Cox *p*-value < 0.05 in the univariate Cox regression analysis were selected irrespective of the direction of the HR and summarized in Supplementary Materials Table S1, including dataset-specific sample sizes, survival endpoints, HRs, 95% CIs, and Cox *p*-values. The complete PrognoScan results are provided in Supplementary Materials Table S2.

### 2.4. Analysis of the Association Between FANCE Expression and Immune Cell Infiltration in LIHC

The immune module of GSCA (https://guolab.wchscu.cn/GSCA/#/immune; accessed on 10 May 2026) was applied to assess the relationships between *FANCE* expression and TIICs in LIHC and other cancer types [39]. Associations between *FANCE* expression and immune cell estimates were evaluated using Spearman’s rank correlation implemented in GSCA. Nominal significance was defined as *p* ≤ 0.05, while FDR ≤ 0.05 indicated significance after multiple-testing correction. Immune cell estimates for diseased and normal tissues generated by algorithms available within the platform were compared. Subsequently, TIMER3 (https://compbio.cn/timer3/; accessed on 1 July 2026) was used for LIHC-specific analyses using multiple immune-deconvolution methods, including XCELL, TIMER, TIDE, ImmuCellAI, EPIC, CIBERSORT, CIBERSORT-ABS, QUANTISEQ, MCP-counter, ABIS, and CONSENSUS_TME, where available. Associations between *FANCE* expression and the immune infiltration estimates generated by these algorithms were assessed using Spearman’s correlation implemented in the TIMER3 Immune_Gene module. The relationships between immune infiltration estimates and OS, PFI, PFS, DSS, and DFI were further evaluated using Cox proportional hazards regression implemented in the Immune-Outcome Module. The platform-reported *p*-values were used without additional correction for multiple immune-cell and survival-endpoint comparisons; therefore, these survival analyses were considered exploratory.

### 2.5. Analysis of FANCE DNA Methylation in LIHC

Methylation levels of the *FANCE* promoter in primary LIHC and normal liver tissues were compared using UALCAN (https://ualcan.path.uab.edu/; accessed on 13 May 2026), with additional comparisons of available clinicopathological characteristics. Statistical differences between groups were evaluated using the unpaired two-sample *t*-test implemented in UALCAN. OncoDB 2.0 (https://oncodb.org/; accessed on 12 May 2026) was used to assess CpG site-specific differences between the tumor and normal tissues. The analysis included 371 LIHC and 41 normal liver samples. The genomic distribution of CpG probes and their relationships with *FANCE* transcript levels and clinicopathological variables were examined via MEXPRESS (https://mexpress.be/; accessed on 13 May 2026) [41]. A total of 23 CpG probes mapped to the *FANCE* genomic region were examined. Correlations between *FANCE* expression and individual CpG methylation levels were evaluated using Pearson correlation, with Benjamini–Hochberg-adjusted *p*-values provided by the platform to account for multiple hypothesis testing. Subsequently, MethSurv (https://biit.cs.ut.ee/methsurv/; accessed on 6 July 2026) was used to determine whether methylation at individual *FANCE*-associated CpG sites was associated with patient survival [42]. Survival associations were evaluated using Cox proportional hazards regression, with HRs and corresponding *p*-values provided by the platform. The four CpG probes presented in the survival analysis comprised cg05261496 and cg03030757, annotated to the 1st exon/5′UTR and CpG island, and cg18744234 and cg08672023, annotated to the gene body and the S_Shore and S_Shelf regions, respectively. No additional multiple-testing correction was applied to the platform-reported *p*-values for these CpG-level survival analyses; therefore, the findings were considered exploratory.

### 2.6. Analysis of FANCE-Associated miRNA and ncRNA Networks in LIHC

Candidate miRNAs associated with *FANCE* were obtained using miRNet 2.0 (https://www.mirnet.ca/; accessed on 13 May 2026) [43]. The resulting gene–miRNA interactions were visualized as a network to identify candidate miRNAs related to *FANCE* using miRNet 2.0 (https://www.mirnet.ca/; accessed on 13 May 2026) [43]. The Encyclopedia of RNA Interactomes (ENCORI; formerly starBase; https://rnasysu.com/encori/; accessed on 13 May 2026) was subsequently used to examine the expression of candidate miRNAs in LIHC, as well as their correlation and prognostic relevance with *FANCE* [44]. Of the miRNAs identified in the network, six candidates were further evaluated for tumor-normal expression, correlation with FANCE expression, and OS. Candidate prioritization was data-driven, with tumor-normal expression, correlation with FANCE expression, and OS used collectively for candidate selection. Based on the outcomes of these analyses, hsa-miR-101-3p was selected for further investigation. Using ENCORI, the lncRNAs, circRNAs, pseudogenes, and sncRNAs associated with hsa-miR-101-3p were identified. Downstream ncRNA candidates were prioritized using the same data-driven approach, based collectively on tumor-normal expression, correlation with hsa-miR-101-3p or *FANCE*, as applicable, and OS. These database-derived relationships were considered putative associations rather than experimentally validated regulatory interactions.

### 2.7. Analysis of FANCE-Associated Drug Response in LIHC

Potential relationships between *FANCE* expression and anticancer treatment outcomes were explored using GEPIA3 (https://gepia3.bioinfoliu.com/drug_survival/; accessed on 13 May 2026) [45]. Associations between *FANCE* expression and survival were examined in patients treated with individual anticancer agents in the available TCGA cohorts. Treatment-annotated cohorts obtained through the drug-survival module were examined to determine whether *FANCE* expression was related to OS following treatment with individual anticancer agents. For each available treatment, OS was examined according to *FANCE* expression in treated and untreated patients, and according to treatment status within the high *FANCE* expression group. ROCplot.org (https://rocplot.com/immune; accessed on 13 May 2026) was used to examine the association between pretreatment *FANCE* expression and response to immune-checkpoint inhibitors in available solid-tumor datasets [46]. Responder and non-responder groups were compared, and ROC analysis was used to evaluate the ability of *FANCE* expression to discriminate treatment response. The analyses included 151 patients for nivolumab (48 responders and 103 non-responders), 257 for pembrolizumab (104 responders and 153 non-responders), and 95 for ipilimumab (45 responders and 50 non-responders). hsa-miR-101-3p expression was additionally evaluated in responder and non-responder groups for available anticancer agents, followed by ROC analysis of treatment response. The analyses included all available tumor types.

### 2.8. Analysis of FANCE–Chemical Interactions in LIHC

Information on chemical–gene interactions was obtained using the Comparative Toxicogenomics Database (CTD; https://ctdbase.org/; accessed on 13 May 2026) [47]. Data on chemicals determined to affect *FANCE* expression were collected, including their CTD chemical identifiers and annotated interaction actions. Chemical similarity information was also examined to identify genes sharing chemical interactions with *FANCE*. Because CTD interaction annotations were compiled from curated experimental reports, these data were treated as qualitative chemical–gene relationships rather than quantitative measures of gene expression changes.

### 2.9. Analysis of Co-Expression and Functional Enrichment Linked with FANCE in LIHC

LinkedOmics (http://www.linkedomics.org/; accessed on 13 May 2026) was used to characterize *FANCE*-associated transcriptomic profiles within the TCGA–LIHC cohort [48]. Genes that were positively or negatively related to *FANCE* expression were ranked according to Pearson correlation coefficients generated by the platform. The top 50 positively and negatively correlated genes were presented as heat maps, and the five genes showing the strongest positive and negative correlations were individually examined. Gene set enrichment analysis (GSEA) in LinkedOmics was applied to the ranked gene set to identify Gene Ontology (GO) biological processes, cellular components, molecular functions, and Kyoto Encyclopedia of Genes and Genomes (KEGG) pathways related to the *FANCE*-associated transcriptional profile [48,49]. GSEA was performed using *p*-value as the rank criterion (signed rank), with a minimum gene-set size of 3 and 500 simulations. Enrichment results were interpreted using the significance criteria provided by the platform, including false discovery rate (FDR)-adjusted values. Complete GSEA results, including normalized enrichment scores (NES) and FDR-adjusted values, are provided in Supplementary Materials Table S3. A separate LinkedOmics-based analysis was conducted using *FANCE* DNA methylation as the molecular feature of interest. Genes positively or negatively related to *FANCE* methylation were identified using Pearson correlation and ranked according to the correlation coefficients generated by the platform. The top 50 positively and negatively correlated genes were presented as heatmaps, and the five genes showing the strongest positive and negative correlations were individually examined. GSEA was conducted using the same GO and KEGG categories.

### 2.10. Analysis of the Prognostic Relevance of FANCE-Associated Genes

Genes identified from the *FANCE* co-expression analysis were further examined using the Survival Map module of Gene Expression Profiling Interactive Analysis 2 (http://gepia2.cancer-pku.cn/; accessed on 13 May 2026) [50]. Positively and negatively associated genes were considered separately, and their relationships with OS and RFS in LIHC were presented as survival maps to determine whether these genes associated with the *FANCE* transcriptional profile also had prognostic relationships within the LIHC cohort.

### 2.11. Analysis of Protein–Protein Interactions and Functional Enrichment Associated with FANCE

The Search Tool for the Retrieval of Interacting Genes/Proteins (STRING; https://string-db.org/; accessed on 9 July 2026) was used to construct a *FANCE* protein–protein interaction (PPI) network [51]. Proteins connected to *FANCE* within the STRING network were functionally characterized. STRING-based enrichment results were examined in terms of their biological processes, cellular components, molecular functions, KEGG pathways, and other functional categories available on the platform. To determine whether the genes encoding the interacting proteins exhibited patterns of coordinated transcription with *FANCE* in LIHC, TIMER3 (https://compbio.cn/timer3/; accessed on 8 July 2026) was used to calculate the gene expression correlations between *FANCE* and the 10 PPI-associated genes identified by STRING.

### 2.12. Statistical Analyses

Statistical results were obtained from the analytical procedures implemented in each database or web-based platform. Statistical tests, effective sample sizes, expression or survival stratification criteria, correlation methods, and other analysis-specific settings are described in the corresponding Methods subsections, where applicable. Survival outcomes were examined using KM analysis and log-rank testing; HRs and 95% CIs were recorded when provided. The Cox proportional hazards model was considered for analyses when implemented by the respective platforms. For analyses in which multiplicity-adjusted statistics were provided by the respective platforms, FDR-adjusted values were used. When only nominal *p*-values were provided, these values were retained and the corresponding findings were interpreted as exploratory. A two-sided *p* value of <0.05 or FDR of ≤0.05 was considered statistically significant, unless otherwise specified by the platform; however, findings based solely on nominal *p*-values without multiple-testing correction were considered exploratory. As several resources draw upon overlapping TCGA–LIHC samples, agreement among database-derived results was considered as cross-platform consistency rather than validation in independent patient populations.

## 3. Results

### 3.1. FANCE mRNA Expression Increases in LIHC

**Figure 1:**
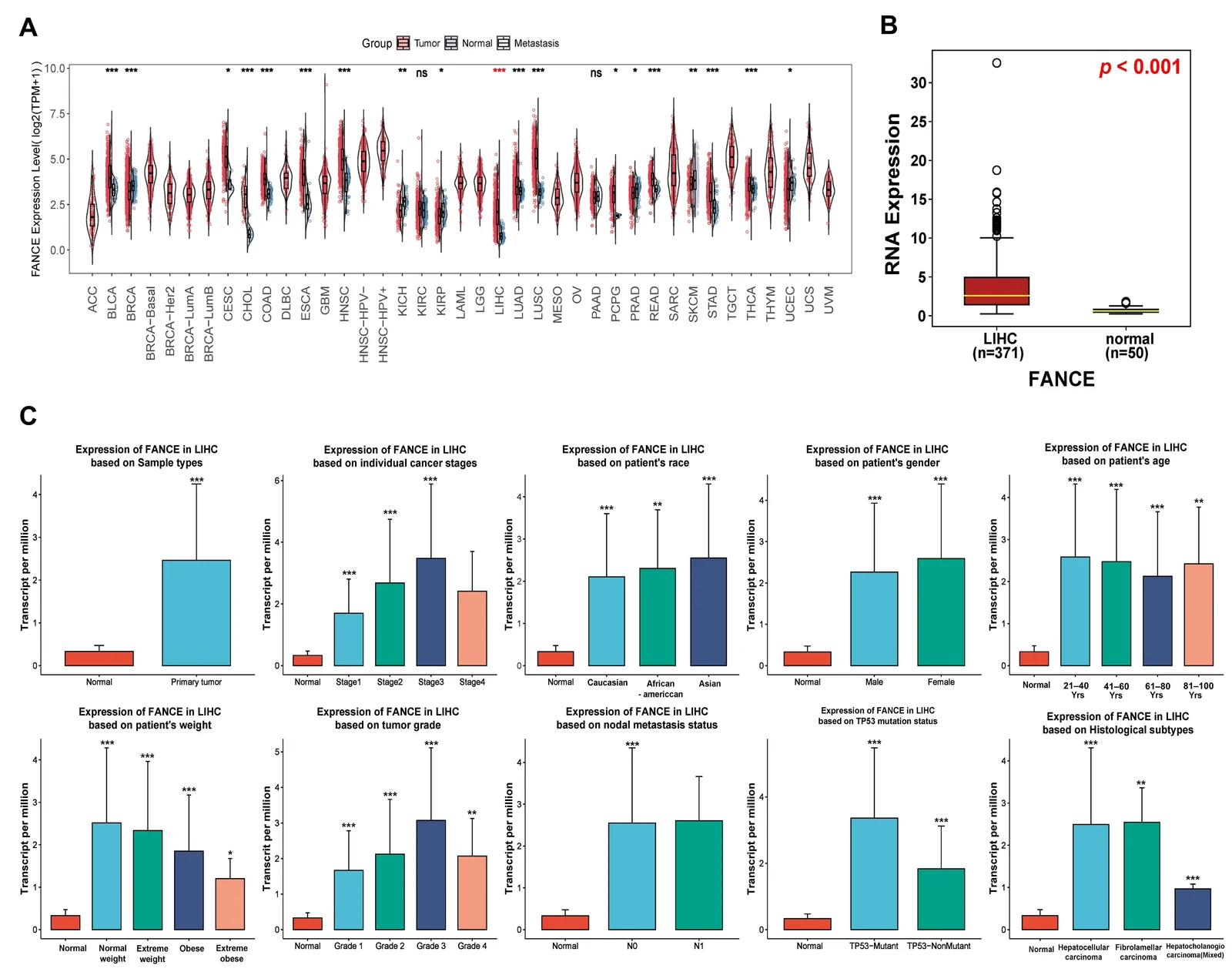
(**A**) Pan-cancer analysis of *FANCE* expression in tumor, normal, and metastatic tissues using the TIMER3 Gene-DE module. (**B**) Comparison of *FANCE* expression in TCGA LIHC (*n* = 371) and normal liver tissue (*n* = 50). (**C**) *FANCE* expression in LIHC according to sample type and clinicopathological characteristics, including race, sex, age, body weight, cancer stage, tumor grade, nodal metastasis status, TP53 mutation status, and histological subtype. Statistical significance is indicated as * *p* < 0.05, ** *p* < 0.01, and *** *p* < 0.001; ns, not significant. The red asterisks highlight the statistical significance observed in LIHC, the primary focus of this study.

*FANCE* mRNA levels were first assessed in tumor and non-tumor tissues for multiple cancers. Significant variations were found within numerous malignancies, including LIHC, in which FANCE expression was higher in tumor than normal liver tissue (Figure 1A). Analysis using OncoDB yielded a similar result, with significantly higher *FANCE* transcript levels in LIHC tissues (*n* = 371) than in normal liver tissues (*n* = 50; *p* < 0.001; Figure 1B). *FANCE* expression was subsequently evaluated according to the clinicopathological characteristics of patients with LIHC. Primary tumors had higher transcript levels than normal tissues, and this was consistent across several patient groups classified by race, sex, age, body weight, cancer stage, tumor grade, nodal status, TP53 mutation status, and histological subtype (Figure 1C).

The differences were not statistically significant for stage 4 or N1 tumors, whereas higher expression was observed in both TP53-mutant and non-mutant tumors and in the major histological subtypes examined (Figure 1C). These analyses reveal elevated *FANCE* transcription in LIHC as well as in several clinicopathological subsets, although they do not indicate a relationship between *FANCE* expression and disease progression.

### 3.2. FANCE Immunohistochemical Staining Intensity Is Higher in LIHC than in Normal Liver Tissues

**Figure 2:**
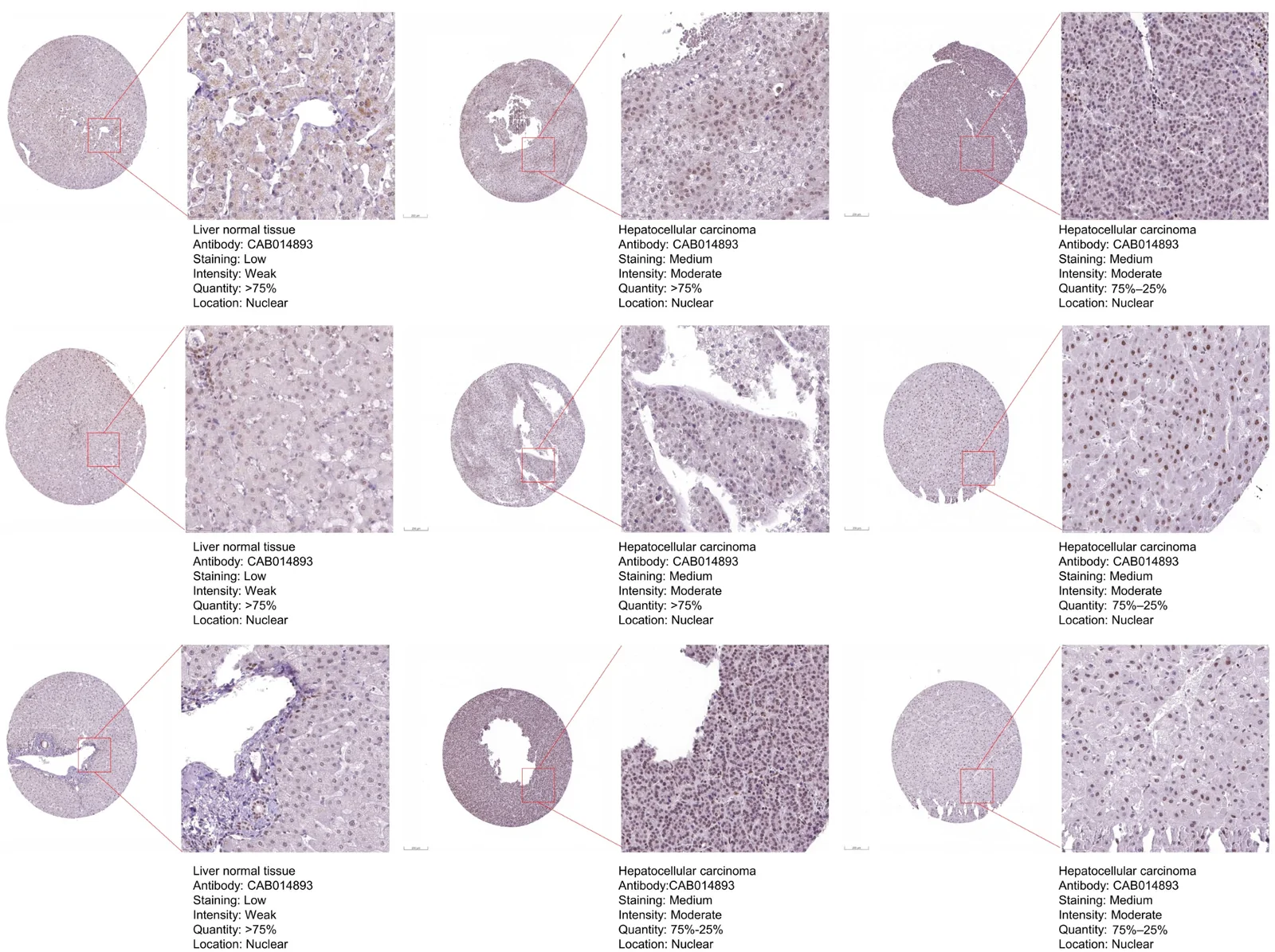
Immunohistochemical analysis of FANCE protein expression in normal liver and LIHC tissues. Representative immunohistochemical staining images of FANCE protein in three normal liver tissues and six LIHC specimens obtained from the Human Protein Atlas (HPA, v25.1). *FANCE* staining was detected in both tissue types, with generally stronger immunoreactivity observed in the available LIHC specimens. Staining was assessed using antibody CAB014893, and the corresponding intensity, quantity, and subcellular localization for each specimen are indicated. Certain regions are enlarged to illustrate the staining pattern.

Immunohistochemical data from HPA were examined to assess FANCE protein staining in normal liver and LIHC tissues. FANCE staining was detected in both tissue types, but the available LIHC specimens generally showed stronger immunoreactivity than normal liver tissues (Figure 2). The staining intensity varied between specimens, indicating a certain degree of tissue-level heterogeneity. Overall, the IHC images showed generally stronger FANCE immunoreactivity in the available LIHC specimens; however, these representative images do not provide a quantitative cohort-level comparison of FANCE protein abundance.

### 3.3. Increased FANCE Expression Is Linked to Unfavorable Survival in LIHC

**Figure 3:**
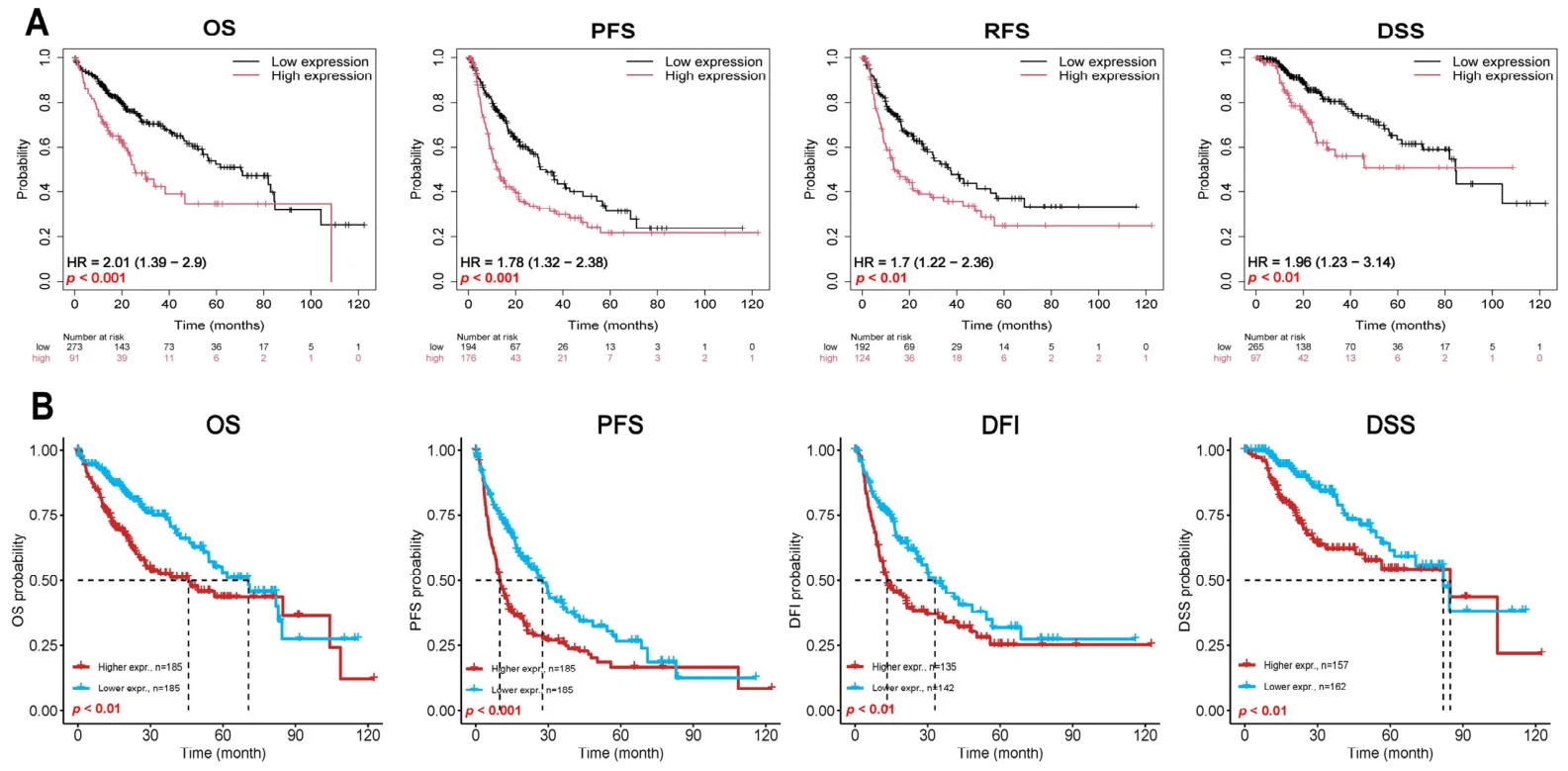
Prognostic significance of *FANCE* expression in LIHC. (**A**) KM plotter analysis of OS, PFS, RFS, and DSS between the high and low *FANCE* expression groups. (**B**) GSCA analysis of OS, PFS, DFI, and DSS of the two groups.

**Figure 4:**
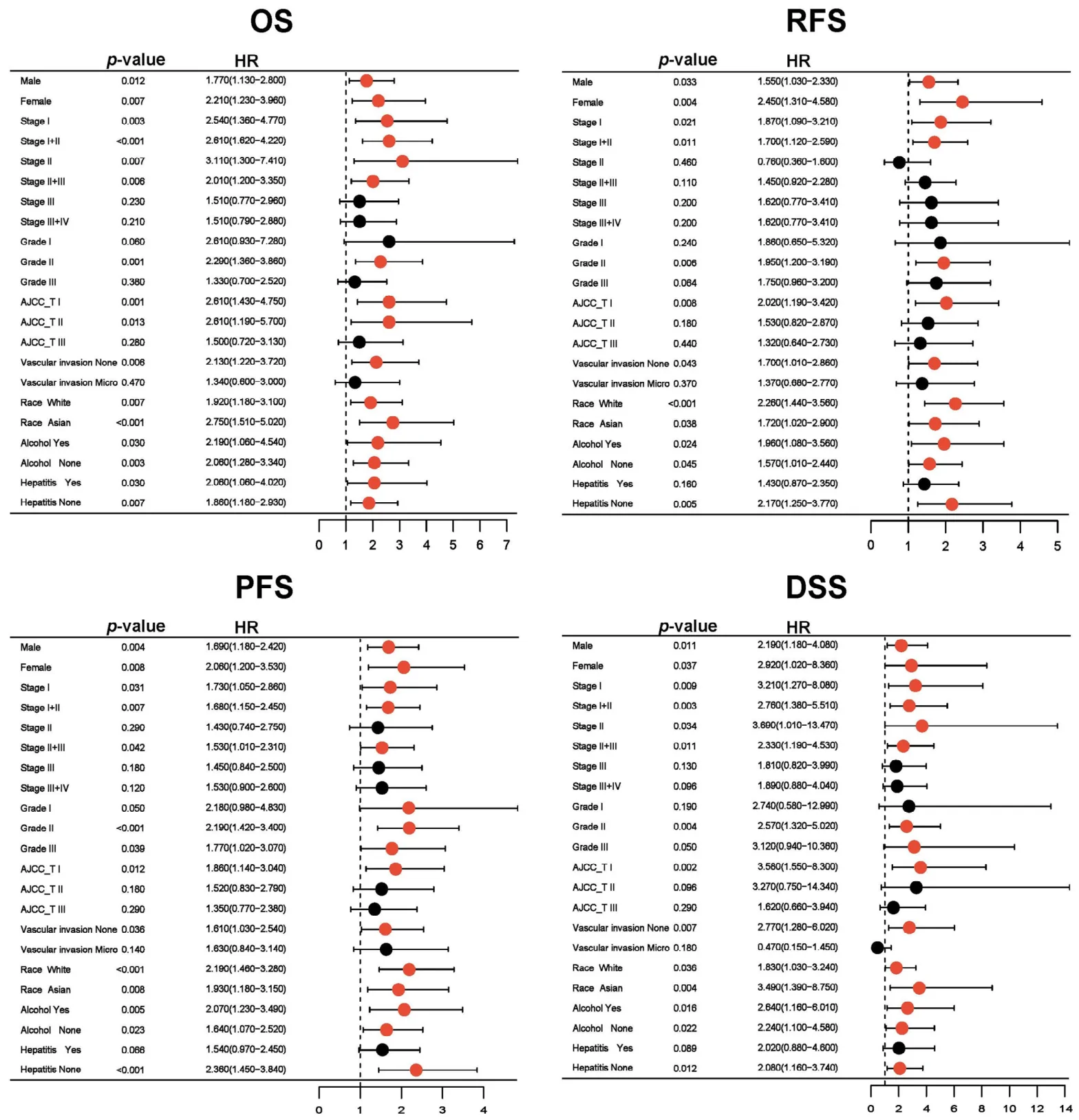
Clinicopathological subgroup survival analyses stratified by *FANCE* expression in LIHC. Forest plots present OS, RFS, PFS, and DSS for the indicated clinicopathological subgroups. Subgroup-specific sample sizes were provided by KM Plotter according to the selected clinicopathological parameters. The reported *p*-values were not adjusted for multiple comparisons, and the subgroup analyses were therefore considered exploratory. Red and black dots indicate statistically significancer and nonsignificant results, respectively.

The prognostic relevance of *FANCE* expression patterns in LIHC was evaluated using multiple survival endpoints. Using the platform-optimized cutoff determined separately for each survival analysis, KM analysis indicated that high *FANCE* expression is associated with shorter OS (HR = 2.01, 95% CI = 1.39–2.90, *p* < 0.001), PFS (HR = 1.78, 95% CI = 1.32–2.38, *p* < 0.001), RFS (HR = 1.70, 95% CI = 1.22–2.36, *p* < 0.01), and DSS (HR = 1.96, 95% CI = 1.23–3.14, *p* < 0.01) (Figure 3A). Using median-based expression stratification, GSCA analysis also yielded comparable findings for OS, PFS, DFI, and DSS (all *p* < 0.01; Figure 3B). In multivariable Cox analysis, *FANCE* expression remained significantly associated with OS after adjustment for age, gender, and tumor stage (adjusted HR = 1.505, 95% CI = 1.239–1.829, *p* < 0.001; *n* = 365). However, when *FANCE* and the proliferation marker *MKI67* were simultaneously included in a multivariable Cox model, the association between *FANCE* expression and OS was attenuated and did not reach statistical significance (HR = 1.361, 95% CI = 0.966–1.919, *p* = 0.0782), whereas *MKI67* remained significantly associated with OS (HR = 1.247, 95% CI = 1.016–1.531, *p* = 0.0348). Survival was further analyzed after stratification based on clinicopathological variables (Figure 4). Higher *FANCE* expression was associated with shorter survival in several clinicopathological subgroups, although the pattern was not consistent in all categories or survival endpoints. Given the multiple subgroup/end-point comparisons and the absence of multiplicity adjustment, these subgroup findings were interpreted as exploratory.

*FANCE* expression was also examined in independent cancer datasets available through PrognoScan to determine whether its prognostic relevance extended beyond LIHC (Table S1). HRs of >1 were reported in several datasets for breast, brain, bladder, lung, skin, and colorectal cancers. The highest HRs were recorded in the GSE9893 breast cancer (HR = 6.66, 95% CI = 2.87–15.47, *p* < 0.001) and the GSE12945 colorectal cancer (HR = 5.67, 95% CI = 1.36–23.72, *p* < 0.05) datasets. Conversely, HRs of <1 were observed in the E-TABM-346 blood cancer, GSE26712 ovarian cancer, HARVARD-LC lung cancer, and GSE3143 breast cancer datasets. These results indicate that the association between *FANCE* expression and survival differed based on cancer types, although it was >1 in most datasets listed in Table S1 (complete results in Table S2). Overall, *FANCE* expression was associated with survival outcomes in LIHC and remained associated with OS after adjustment for age, gender, and tumor stage, although this association was attenuated when *MKI67* was included in the model.

### 3.4. FANCE Expression Is Related to Immune Cell Infiltration in LIHC

The relationship between *FANCE* expression and immune cell infiltration was first assessed using GSCA. Correlation patterns differed considerably according to cancer type and immune cell population (Figure S1). In LIHC, *FANCE* expression was significantly correlated with several immune cell estimates, including B cell, T cell, macrophage, dendritic cell, and other myeloid and lymphoid populations. Both positive and negative correlations were detected, indicating that the immune profile associated with *FANCE* expression is not restricted to a single immune cell lineage. Immune infiltration in LIHC was further evaluated by applying multiple computational algorithms (Table 1). TIMER analysis showed a positive correlation between *FANCE* expression and B cells (R = 0.325, *p* < 0.001), myeloid dendritic cells (R = 0.489, *p* < 0.001), macrophages (R = 0.388, *p* < 0.001), neutrophils (R = 0.222, *p* < 0.001), CD4+ T cells (R = 0.241, *p* < 0.001), and CD8+ T cells (R = 0.150, *p* < 0.01). Results from the different deconvolution methods were partly consistent but not uniform. CD8+ T-cell estimates were positively correlated with FANCE expression in the algorithms providing these estimates. Positive correlations were also found for B-cell estimates in multiple algorithms, with some differences according to B-cell subtype. Negative correlations were detected for different immune-cell estimates depending on the deconvolution method. For some cell populations, the direction of the correlation also differed between algorithms, particularly for macrophage-related estimates. For example, macrophage estimates were positively correlated with *FANCE* expression in TIMER and ImmuCellAl but negatively correlated in xCell and EPIC. M2 macrophage estimates were also negative in xCell and CIBERSORT but positive in CIBERSORT-ABS and quanTIseq.

The clinical relevance of immune cell estimates identified in these analyses was subsequently assessed using survival endpoints (Table 2). Significant associations with survival were identified in several immune cell populations. Both negative and positive Z-scores were observed depending on the immune cell population and survival endpoint. Macrophage-related estimates were not uniform among the deconvolution methods; TIMER- and ImmunCellAl-derived estimates had positive Z-scores for several endpoints, whereas EPIC-derived macrophage estimates had negative Z-scores. These findings indicate that several immune cell estimates correlated with *FANCE* expression were also associated with survival outcomes in LIHC; however, the direction of these trends varied by immune cell population and the analytical method used.

### 3.5. FANCE Promoter Methylation Is Reduced in LIHC

**Figure 5:**
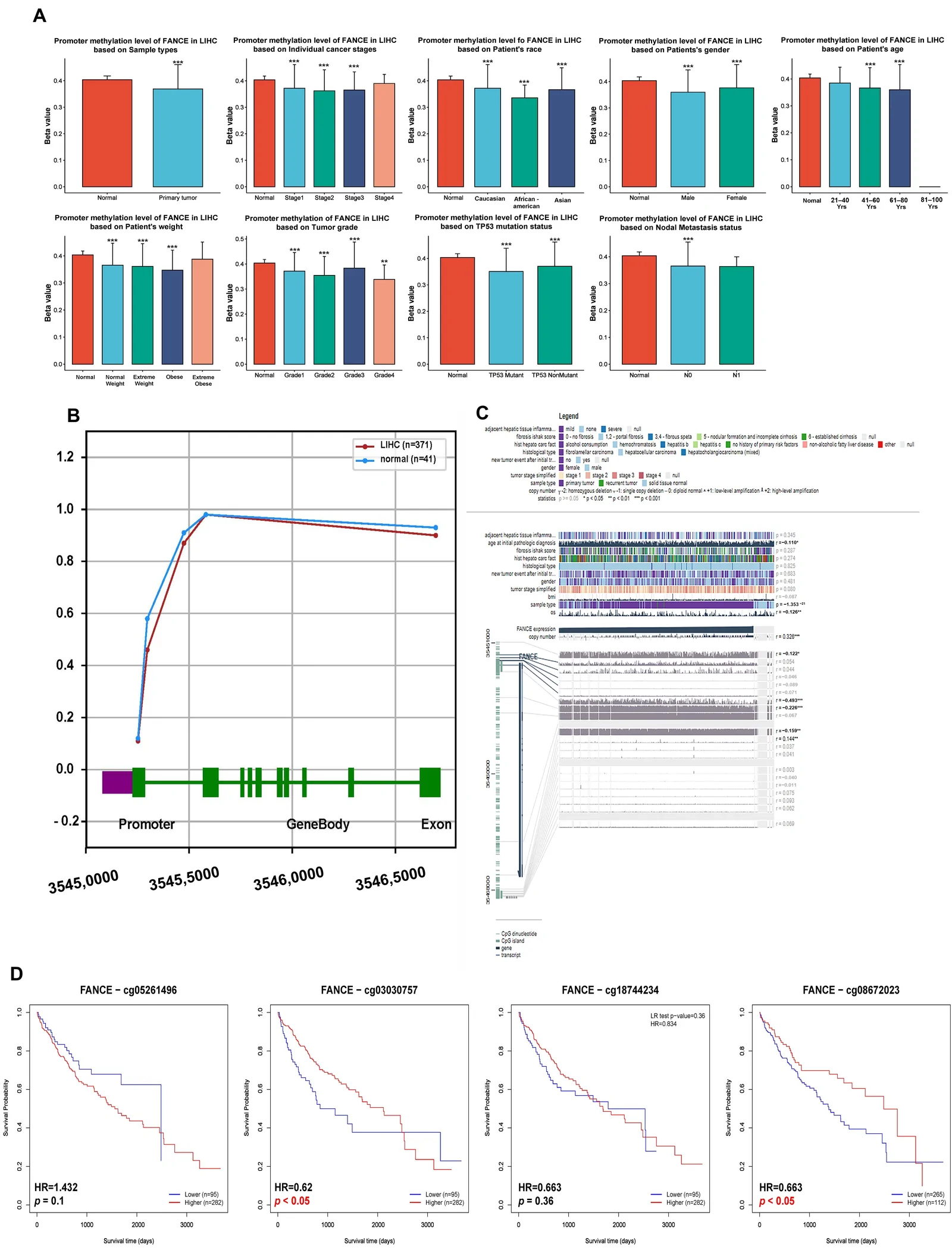
DNA methylation profile of *FANCE* in LIHC. (**A**) *FANCE* promoter methylation levels in LIHC and normal liver tissues and in clinicopathological subgroups detected using UALCAN. (**B**) Site-specific methylation profiles within the *FANCE* locus in LIHC and normal liver tissues. (**C**) MEXPRESS analysis showing *FANCE* expression, CpG methylation, and clinical and molecular features in LIHC. The truncated labels refer to adjacent hepatic tissue inflammation and new tumor event after initial treatment. (**D**) KM survival analyses for select *FANCE* CpG sites estimated using MethSurv. * *p* < 0.05, ** *p* < 0.01, and *** *p* < 0.001.

Methylation of *FANCE* DNA was examined to establish its epigenetic profile in LIHC. Promoter methylation was significantly lower in primary LIHC tissues than in normal liver tissue (Figure 5A). Reduced methylation was also detected in several clinicopathological groups classified according to race, sex, age, body weight, tumor stage, tumor grade, TP53 mutation status, and nodal status. The variations were not statistically significant in some categories, including the stage 4 and N1 groups, indicating that the methylation pattern was not uniform across all clinical subsets. DNA methylation distribution along the *FANCE* genomic region was further examined in LIHC and normal liver tissues (Figure 5B). Methylation levels varied per genomic position, with relatively elevated levels detected in some portions of the promoter and gene body. Analysis using *FANCE* expression as well as DNA methylation and clinical information also identified multiple CpG sites within the *FANCE* locus with different methylation profiles (Figure 5C). The strongest negative correlation with *FANCE* expression was observed for cg03030757 (Pearson *r* = −0.493, BH-adjusted *p* = 1.46 × 10^−22^), followed by cg18744234 (*r* = −0.226, BH-adjusted *p* = 3.45 × 10^−6^). The prognostic relevance of individual *FANCE* CpG sites was assessed using methylation-based survival analysis (Figure 5D). Methylation at cg03030757 (HR = 0.62, *p* < 0.05) and cg08672023 (HR = 0.663, *p* < 0.05) was linked to variations in survival, whereas that at cg05261496 (HR = 1.432, *p* = 0.1) and cg18744234 (HR = 0.663, *p* = 0.36) did not reach statistical significance. Thus, the data indicate lower *FANCE* promoter methylation levels in LIHC and identify specific CpG sites with potential prognostic relevance, although the functional consequences of these alterations cannot be determined from the present analyses.

### 3.6. Reduced miR-101-3p Expression Is Correlated with FANCE Expression and Survival in LIHC

**Figure 6:**
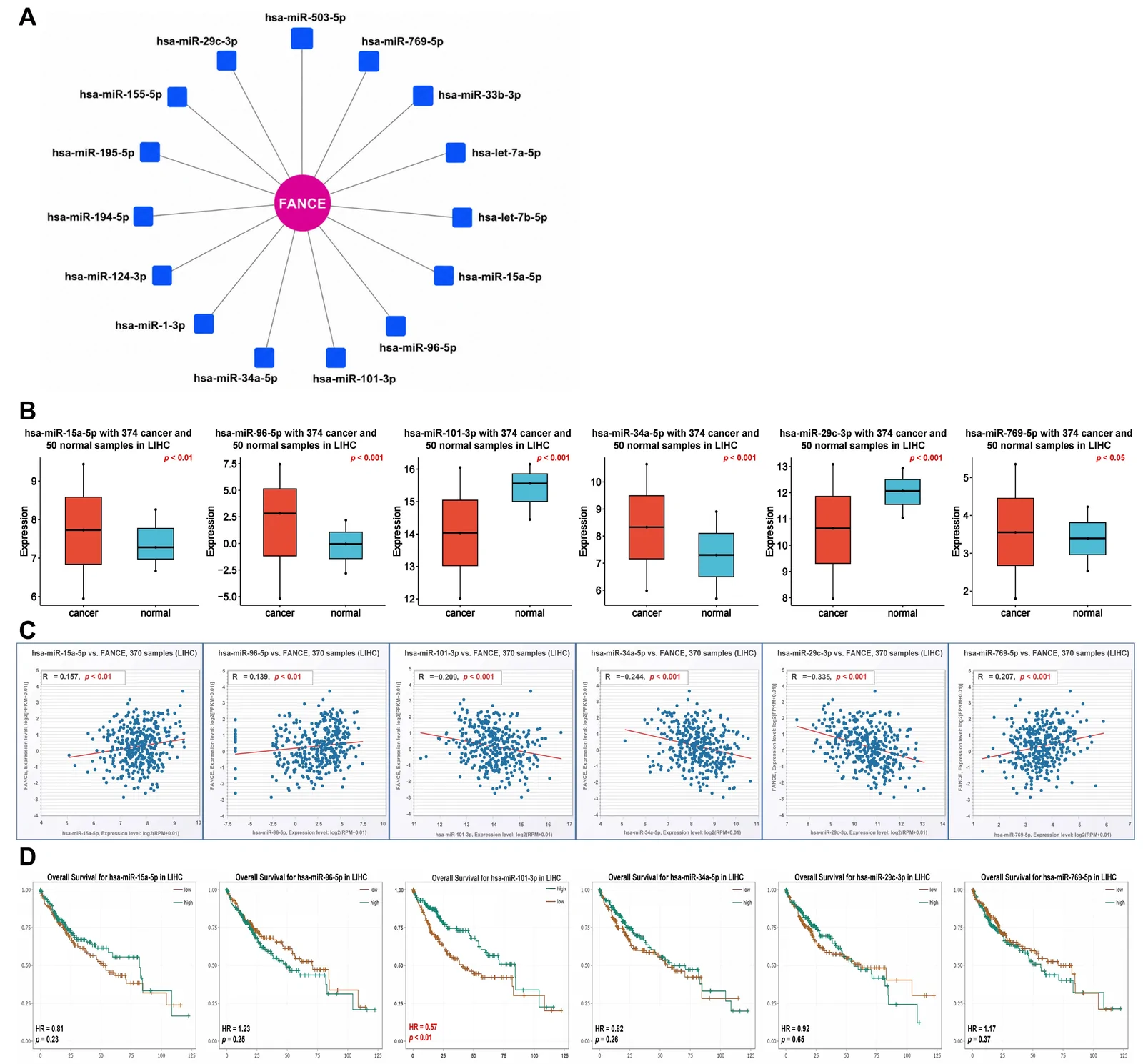
Identification and evaluation of candidate miRNAs related to *FANCE* in LIHC. (**A**) Predicted *FANCE*–miRNA interaction network generated using miRNet. (**B**) Expression profiles of select candidate miRNAs in LIHC and normal liver tissue. (**C**) Correlations between candidate miRNAs and *FANCE* expression in LIHC. (**D**) Analyses of OS associated with the select candidate miRNAs.

**Figure 7:**
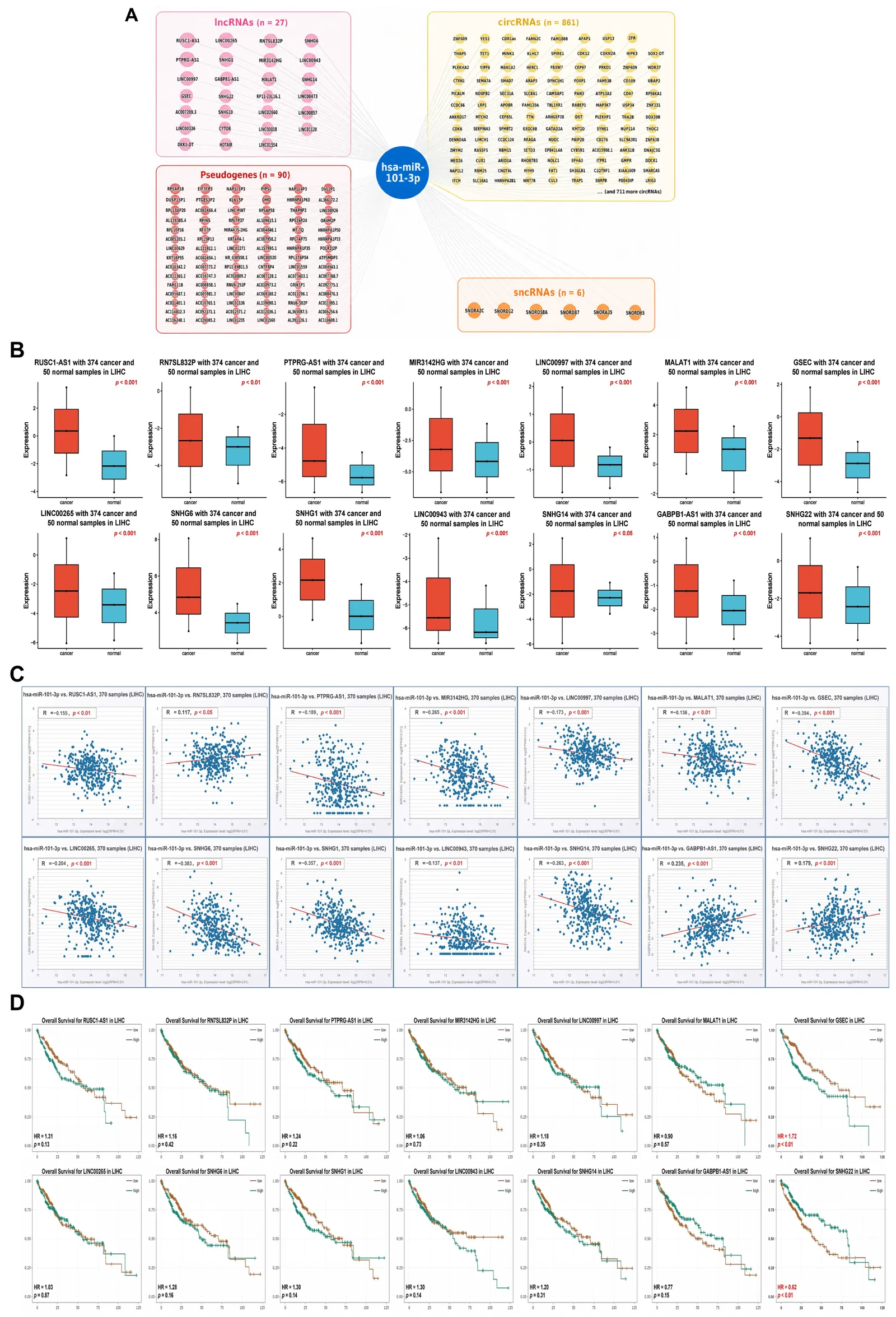
hsa-miR-101-3p-related ncRNA interaction network and select long-RNA candidates in LIHC. (**A**) Predicted ncRNA interaction network centered on hsa-miR-101-3p, comprising lncRNAs, circRNAs, pseudogenes, and sncRNAs. Of the 861 circRNAs identified, 150 are displayed for clarity, while the remaining 711 are omitted from the visualization. (**B**) Expression profiles of specific candidate long-RNAs in LIHC and normal liver tissue. (**C**) Correlations between select transcripts and hsa-miR-101-3p expression in LIHC. (**D**) OS analyses for specific long-RNA candidates.

Potential *FANCE*-linked miRNAs were identified through network-based analysis (Figure 6A). Fourteen candidate miRNAs were identified, including hsa-miR-15a-5p, hsa-miR-96-5p, hsa-miR-101-3p, hsa-miR-34a-5p, hsa-miR-29c-3p, hsa-miR-769-5p, hsa-miR-33b-3p, hsa-let-7a-5p, hsa-let-7b-5p, hsa-miR-124a-3p, hsa-miR-1-3p, hsa-miR-194-5p, hsa-miR-195-5p, and hsa-miR-155-5p. Of these, six were further evaluated in terms of their expression patterns in LIHC, correlations with *FANCE*, and survival data (Figure 7B–D). Compared with normal liver tissues, the expression of hsa-miR-15a-5p, hsa-miR-96-5p, hsa-miR-34a-5p, and hsa-miR-769-5p was higher in LIHC, whereas that of hsa-miR-101-3p and hsa-miR-29c-3p was lower (Figure 6B). Correlation analysis revealed that *FANCE* was positively correlated with hsa-miR-15a-5p (R = 0.157, *p* < 0.01), hsa-miR-96-5p (R = 0.139, *p* < 0.01), and hsa-miR-769-5p (R = 0.207, *p* < 0.001) and negatively with hsa-miR-101-3p (R = −0.209, *p* < 0.001), hsa-miR-34a-5p (R = −0.244, *p* < 0.001), and hsa-miR-29c-3p (R = −0.335, *p* < 0.001) (Figure 6C).

Among the six miRNAs, only hsa-miR-101-3p showed a statistically significant difference in OS (HR = 0.57, *p* < 0.01; Figure 7D), although the relation with hsa-miR-29c-3p showed borderline significance (HR = 0.82, *p* = 0.065). Taken together, the expression of hsa-miR-101-3p decreased in LIHC, was negatively correlated with *FANCE* expression, and was significantly associated with OS, supporting its prioritization for further investigation. These bioinformatic findings, however, do not establish direct miRNA-mediated regulation of *FANCE*.

### 3.7. miR-101-3p-Related ncRNAs Show Distinct Expression, Correlation, and Survival Patterns in LIHC

To characterize the ncRNA network linked to hsa-miR-101-3p, potential interacting transcripts were identified and classified per RNA type. The network obtained contained 27 lncRNAs, 861 circRNAs, 90 pseudogenes, and 6 sncRNAs (Figure 7A). Select candidates from these RNA classes were subsequently evaluated via expression, correlation, and survival analyses; 14 long-RNA candidates were examined in greater detail (Figure 7B–D). Most were more highly expressed in LIHC than in normal liver tissue (Figure 7B). RUSC1-AS1, PTPRG-AS1, MIR3142HG, LINC00997, MALAT1, GSEC, LINC00265, SNHG6, SNHG1, LINC00943, GABPB1-AS1, and SNHG22 differed significantly between tumor and normal tissues at *p* < 0.001, and significant differences were also found for RN7SL832P and SNHG14. Correlation analysis identified an inverse relationship between hsa-miR-101-3p and several of these transcripts, including RUSC1-AS1, PTPRG-AS1, MIR3142HG, LINC00997, MALAT1, GSEC, LINC00265, SNHG6, SNHG1, LINC00943, and SNHG14 (Figure 7C). GSEC had the strongest negative correlation (R = −0.394, *p* < 0.001). In contrast, RN7SL832P, GABPB1-AS1, and SNHG22 were positively correlated with hsa-miR-101-3p. KM analyses identified significant OS variations for GSEC (HR = 1.72, *p* < 0.01) and SNHG22 (HR = 0.62, *p* < 0.01), whereas the other candidates did not achieve statistical significance (Figure 7D).

**Figure 8:**
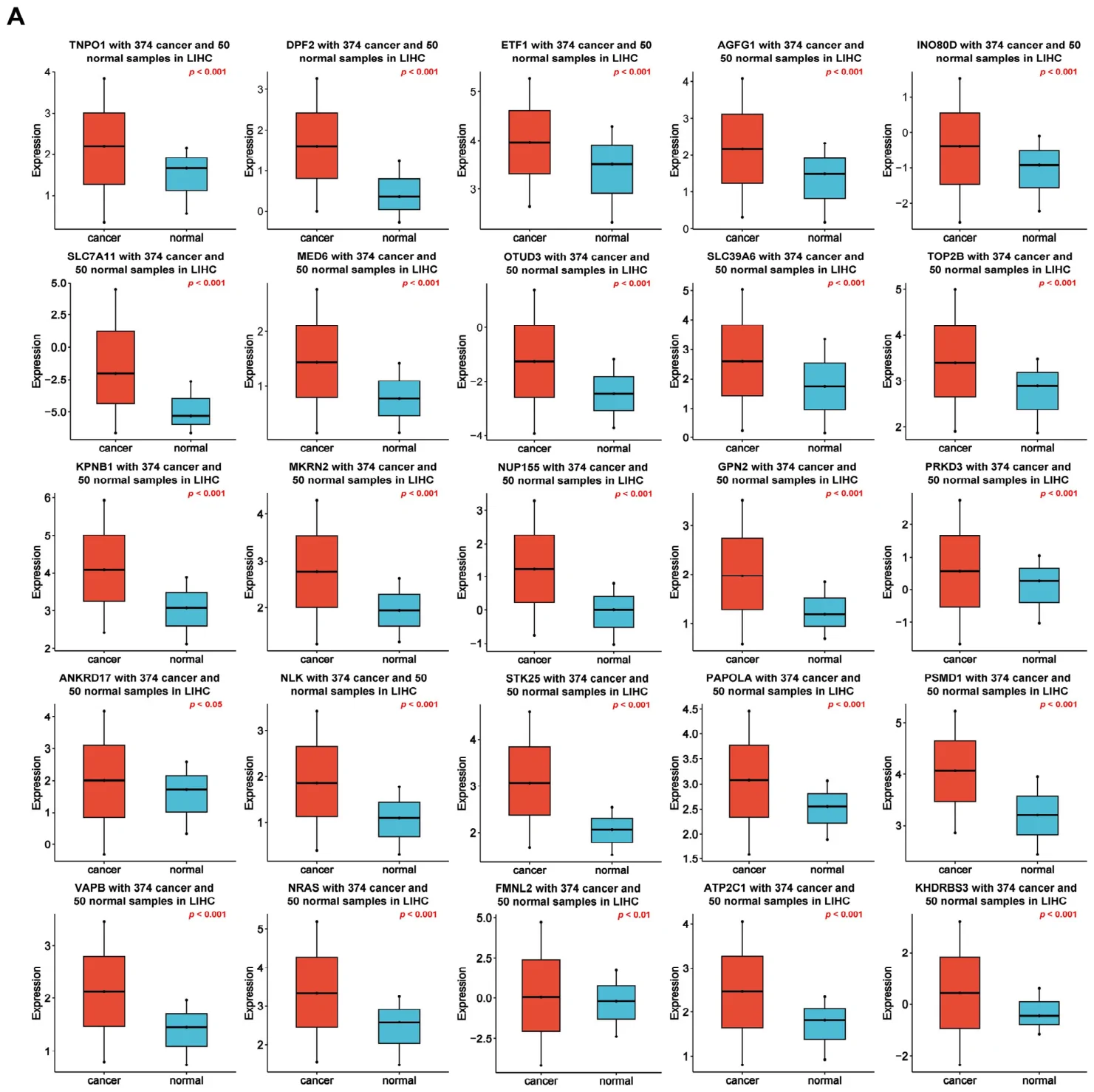

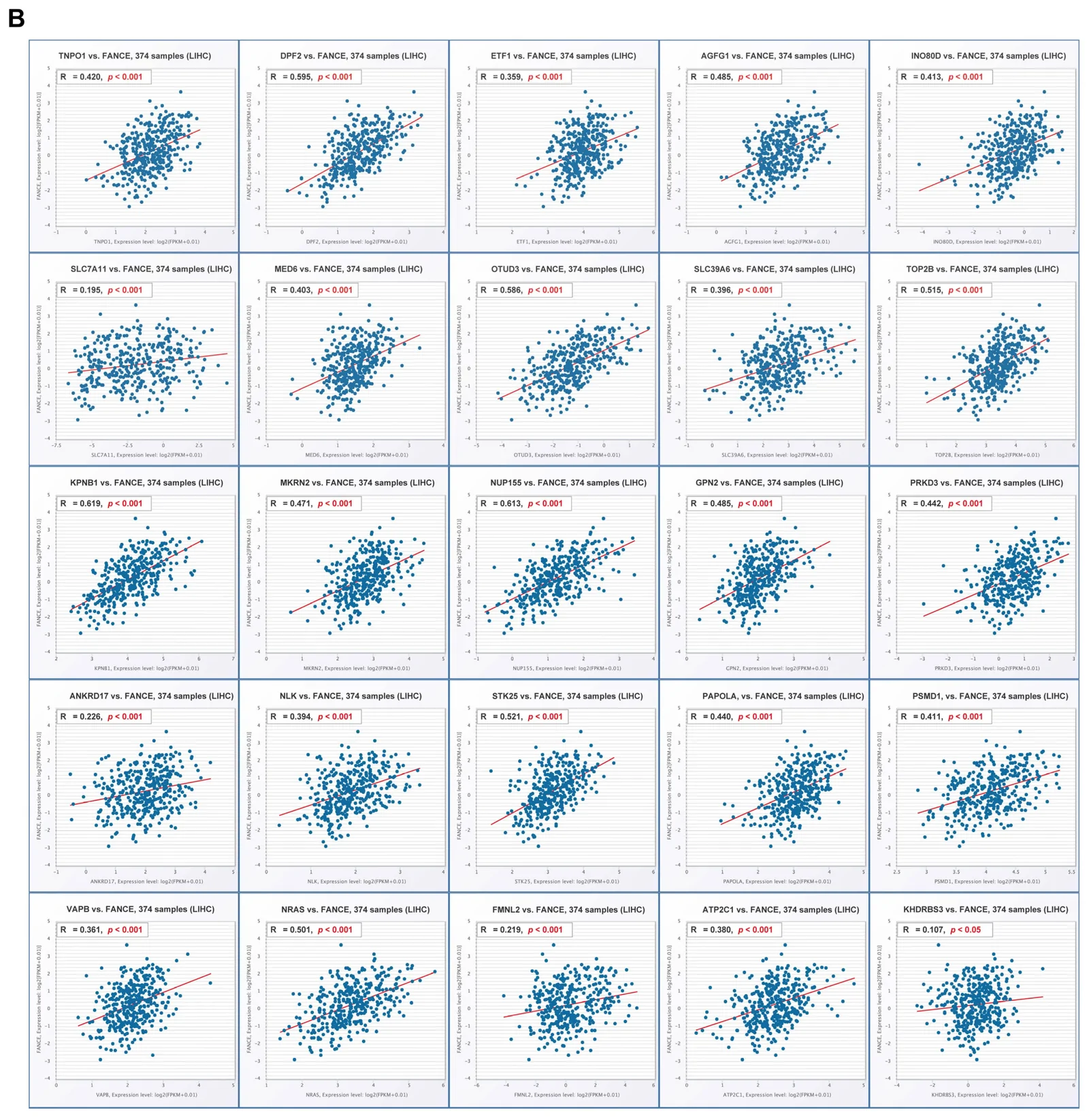

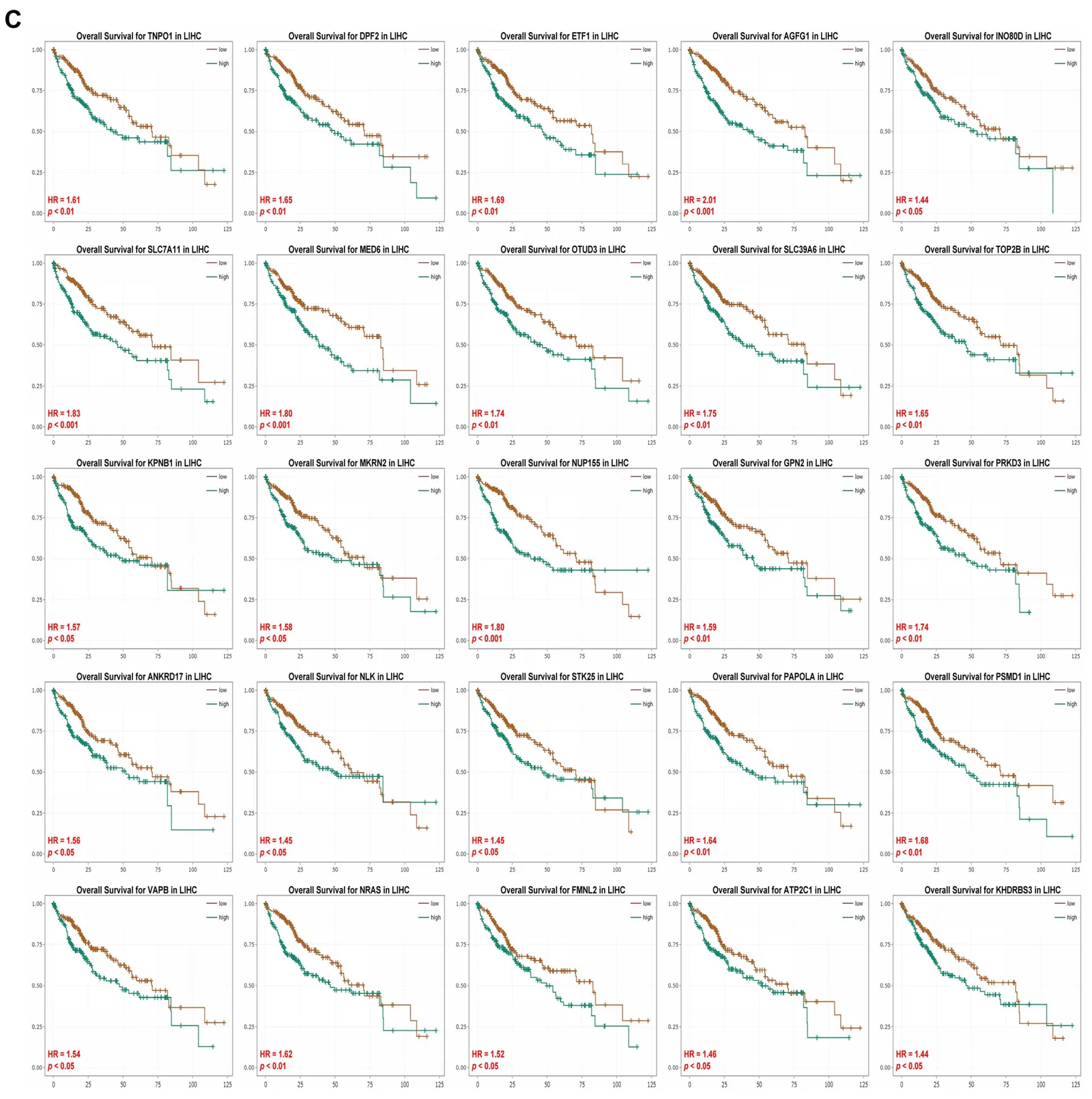
Expression and clinical relevance of circRNA-associated genes related to hsa-miR-101-3p in LIHC. (**A**) Gene-level expression profiles of select circRNA-associated genes in normal liver and LIHC tissues. (**B**) Correlations between the select genes and *FANCE* expression in LIHC. (**C**) OS curves for the select genes. The *x*-axis indicates survival time (in months), and the *y*-axis represents survival probability. The expression data represent gene-level measurements associated with circRNA records and do not directly quantify individual circRNA isoforms.

The circRNA component of the hsa-miR-101-3p network was further examined using gene-level data obtained from the downstream analytical platform. Twenty-five genes linked to the circRNA records demonstrated significantly greater expression in LIHC than in normal liver tissue (Figure 8A). However, these measurements should not be interpreted as direct assessments of individual circular RNA isoforms as they were derived from gene-level expression data. All 25 genes were positively correlated with *FANCE* expression in LIHC (Figure 8B). Relatively robust correlations were found for KPNB1 (R = 0.619), NUP155 (R = 0.613), DPF2 (R = 0.595), and OTUD3 (R = 0.586) (all *p* < 0.001). KM analysis further revealed a shorter OS in the high-expression groups for each of the 25 examined genes (Figure 8C).

**Figure 9:**
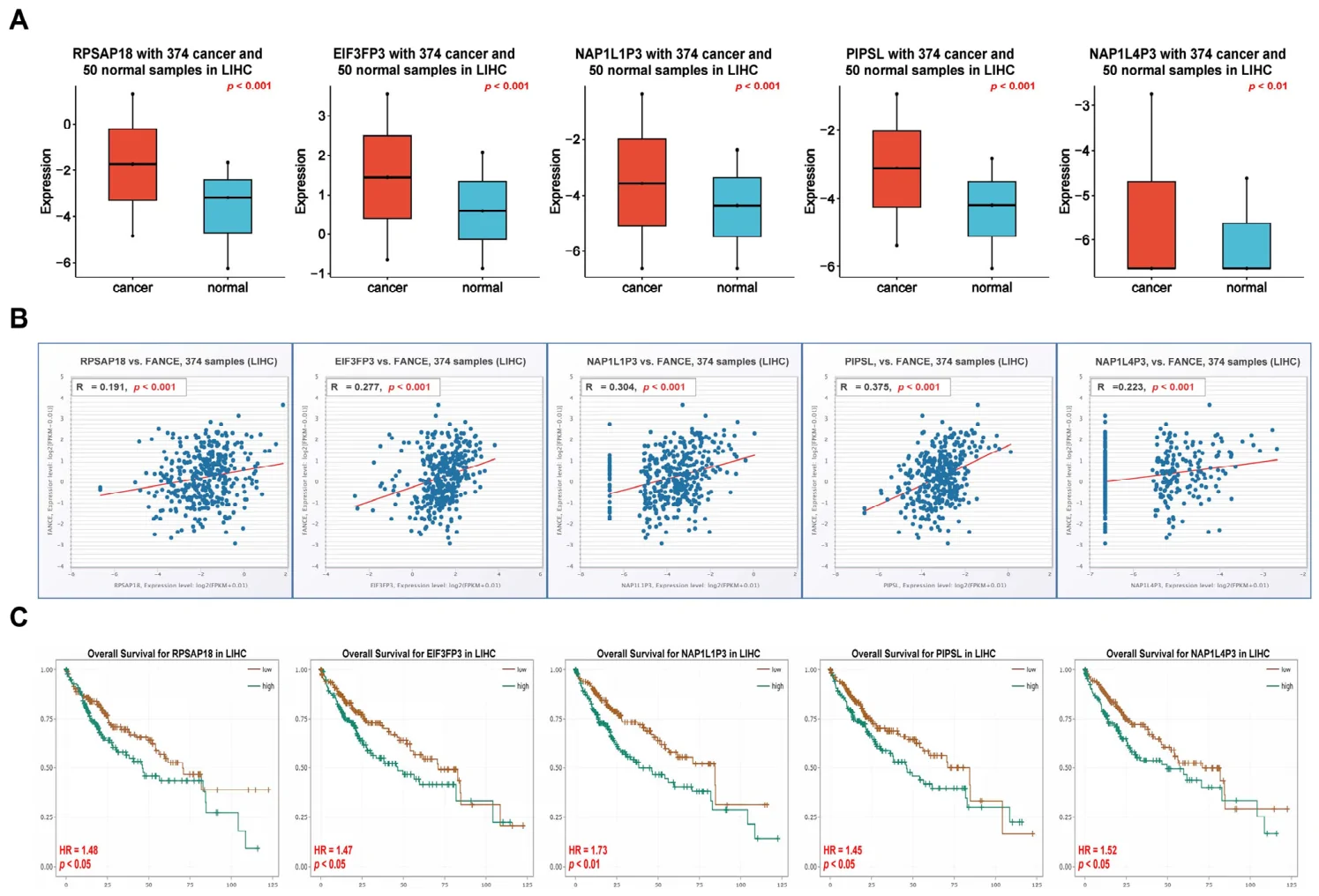
Expression and clinical relevance of select hsa-miR-101-3p-associated pseudogenes in LIHC. (**A**) Expression profiles of the selected pseudogenes in LIHC and normal liver tissue. (**B**) Correlations between the selected pseudogenes and *FANCE* expression in LIHC. (**C**) OS curves for the pseudogenes. The *x*-axis represents survival time (in months), and the *y*-axis indicates survival probability.

To further investigate the pseudogene component of the hsa-miR-101-3p network, 90 candidate pseudogenes were screened based on their expression, correlation with *FANCE*, and prognostic significance. Among these, five pseudogenes identified from the hsa-miR-101-3p-related network—*RPSAP18*, *EIF3FP3*, *NAP1L1P3*, *PIPSL*, and *NAP1L4P3*—were assessed based on the same analytical framework. Expression of all five genes was higher in LIHC than in normal liver tissues, with *p* < 0.001 for *RPSAP18*, *EIF3FP3*, *NAP1L1P3*, and *PIPSL* and *p* < 0.01 for *NAP1L4P3* (Figure 9A). Each pseudogene correlated positively with *FANCE* expression (Figure 9B), with coefficients ranging from 0.191 for *RPSAP18* to 0.375 for *PIPSL* (all *p* < 0.001). Higher expression of each pseudogene was also linked to shorter OS, with HRs of 1.48 for *RPSAP18*, 1.47 for *EIF3FP3*, 1.73 for *NAP1L1P3*, 1.45 for *PIPSL*, and 1.52 for *NAP1L4P3* (all *p* < 0.05; Figure 9C).

**Figure 10:**
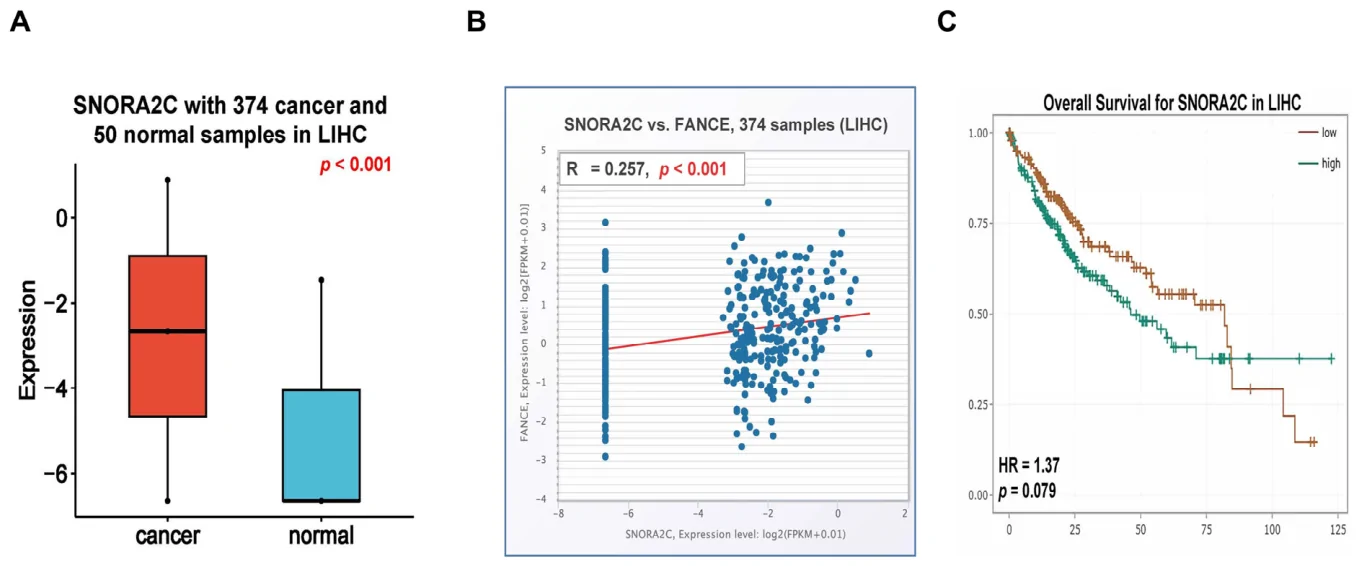
Expression and clinical relevance of the hsa-miR-101-3p-associated small ncRNA candidate SNORA2C in LIHC. (**A**) Expression profile of SNORA2C in LIHC and normal liver tissue. (**B**) Correlation between SNORA2C and *FANCE* expression in LIHC. (**C**) OS curve related to SNORA2C levels. The *x*-axis indicates survival time (months), and the *y*-axis represents survival probability.

Of the sncRNAs identified within the network, SNORA2C was further evaluated for its expression, correlation with *FANCE*, and survival pattern (Figure 10). SNORA2C expression was significantly higher in LIHC than in normal liver tissues (*p* < 0.001; Figure 10A) and positively correlated with *FANCE* expression (R = 0.257, *p* < 0.001; Figure 10B). Patients with high SNORA2C expression tended to have shorter OS, but the difference was not statistically significant (HR = 1.37, *p* = 0.079; Figure 10C). Thus, the expression of SNORA2C was altered and positively correlated with *FANCE*, and its association with survival remained inconclusive.

### 3.8. FANCE and hsa-miR-101-3p Show Drug-Dependent Patterns in Treatment-Response Analyses

**Figure 11:**
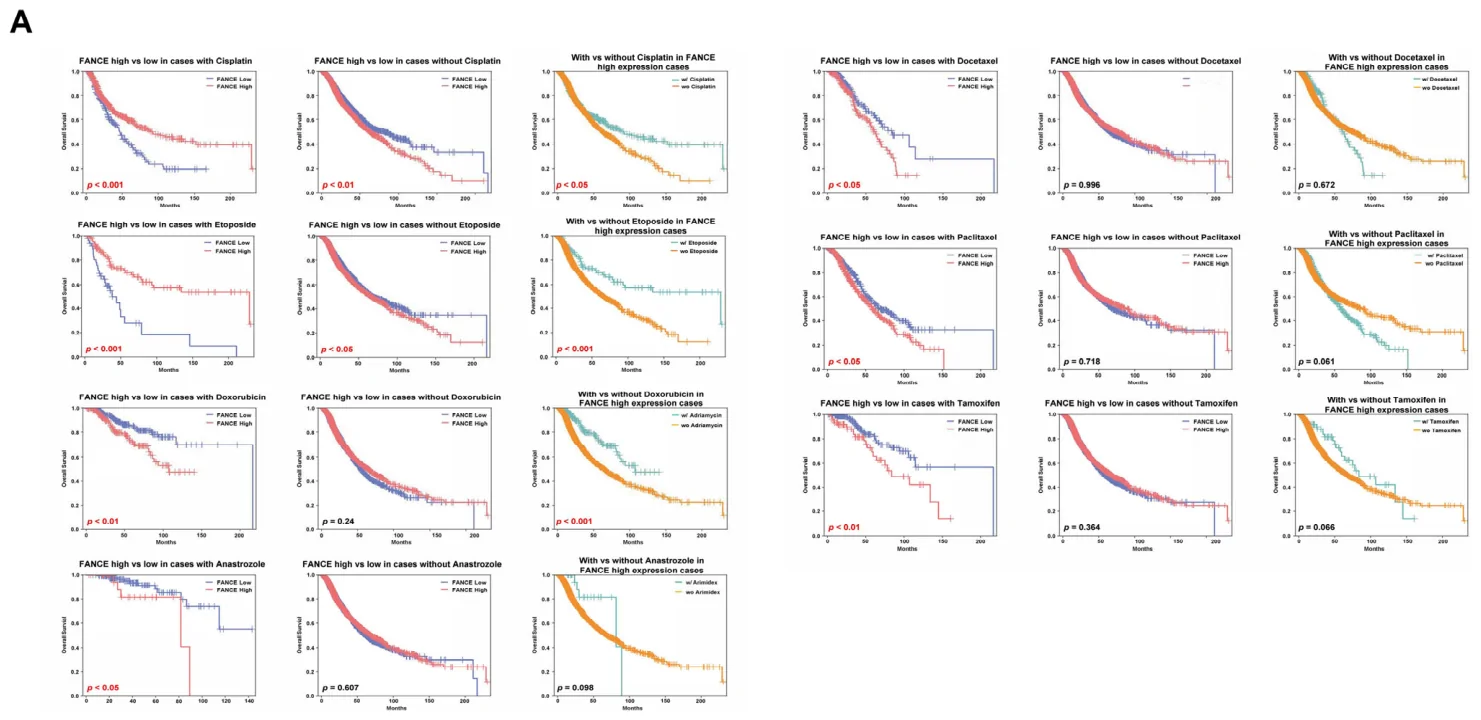

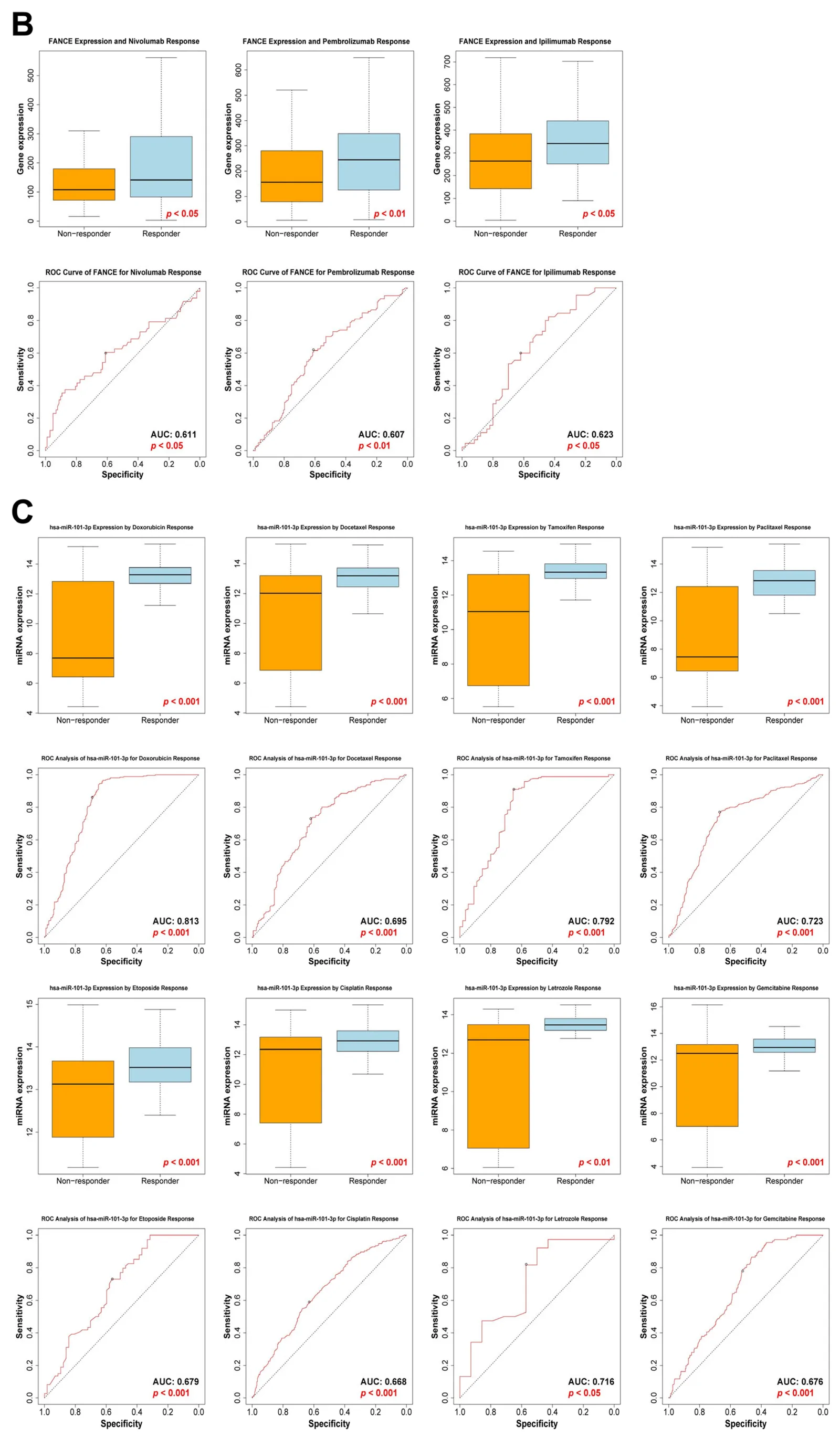
Treatment-response associations of *FANCE* and hsa-miR-101-3p expression in external cancer cohorts. (**A**) OS curves stratified by *FANCE* expression and treatment with cisplatin, etoposide, doxorubicin, anastrozole, docetaxel, paclitaxel, and tamoxifen. For each agent, survival was evaluated based on *FANCE* expression in the treated and untreated groups, along with treatment within the high-*FANCE* subgroup. The corresponding sample sizes for the drug/non-drug groups were 774/2614, 151/3237, 472/2916, 174/3214, 398/2990, 832/2556, and 296/3092, respectively. (**B**) *FANCE* expression in responders and non-responders to nivolumab, pembrolizumab, and ipilimumab, with ROC analyses of the treatment response. The corresponding sample sizes were 151 (48 responders and 103 non-responders), 257 (104 responders and 153 non-responders), and 95 (45 responders and 50 non-responders), respectively. (**C**) hsa-miR-101-3p expression in responders and non-responders to doxorubicin, docetaxel, tamoxifen, paclitaxel, etoposide, cisplatin, letrozole, and gemcitabine, with ROC analyses of treatment response. Response was defined according to relapse-free survival at 12 months. The corresponding sample sizes were 474 (257 responders and 217 non-responders), 337 (191 responders and 146 non-responders), 221 (166 responders and 55 non-responders), 809 (355 responders and 454 non-responders), 131 (74 responders and 57 non-responders), 710 (391 responders and 319 non-responders), 52 (38 responders and 14 non-responders), and 416 (111 responders and 305 non-responders), respectively. AUC, area under the receiver operating characteristic curve; OS, overall survival; ROC, receiver operating characteristic.

The potential therapeutic relevance of *FANCE* was examined in treatment-annotated survival cohorts (Figure 11A). The relationship between *FANCE* expression and OS differed according to the administered drug. In cisplatin-treated patients, the high *FANCE* expression group had better survival than the low-expression group (*p* < 0.001). A similar pattern was noted in etoposide-treated patients (*p* < 0.001). Survival also varied between treated and untreated patients within the high-*FANCE* group for cisplatin (*p* < 0.05) and etoposide (*p* < 0.001). Other agents, including docetaxel, paclitaxel, doxorubicin, tamoxifen, and anastrozole, showed variable survival patterns according to *FANCE* expression and treatment status.

These findings indicate that the survival pattern related to *FANCE* expression differed according to treatment context rather than following a uniform direction. As the analyses involved heterogeneous treatment-annotated cohorts across cancer types, were not matched for potential clinical confounders, and did not include formal testing of expression-by-treatment interactions, they do not establish *FANCE* as a predictive marker of drug response in LIHC. *FANCE* expression was subsequently evaluated in patients receiving immune checkpoint inhibitors (Figure 11B). *FANCE* expression was higher in responders to nivolumab, pembrolizumab, and ipilimumab than in non-responders (*p* < 0.05, *p* < 0.01, and *p* < 0.05, respectively). The analyses included 151 patients for nivolumab (48 responders and 103 non-responders), 257 for pembrolizumab (104 responders and 153 non-responders), and 95 for ipilimumab (45 responders and 50 non-responders). ROC analysis yielded AUC values of 0.611 for nivolumab, 0.607 for pembrolizumab, and 0.623 for ipilimumab. Thus, although expression was significantly different between response groups, the AUC values indicated limited discriminatory performance of *FANCE* alone.

Treatment response was also analyzed for hsa-miR-101-3p with available drug-response cohorts (Figure 11C). Treatment response was defined as relapse-free survival at 12 months, and all available tumor types were included. Higher hsa-miR-101-3p expression was observed in responders for doxorubicin, docetaxel, tamoxifen, paclitaxel, etoposide, cisplatin, letrozole, and gemcitabine. The corresponding sample sizes (responders/non-responders) were 257/217 for doxorubicin, 191/146 for docetaxel,166/55 for tamoxifen, 355/454 for paclitaxel, 74/57 for etoposide, 391/319 for cisplatin, 38/14 for letrozole, and 111/305 for gemcitabine. ROC analysis produced AUC values of 0.813, 0.695, 0.792, 0.723, 0.679, 0.668, 0.716, and 0.676, respectively. These response-related patterns were derived from heterogeneous pan-cancer cohorts and should not be regarded as LIHC-specific predictive evidence.

### 3.9. FANCE Shares Chemical Interaction Profiles with Various Genes

Chemical–gene interaction data for *FANCE* were retrieved from the CTD. Twenty chemicals were identified with records indicating increased *FANCE* expression (Table 3). These included pharmacologically relevant compounds such as cyclosporine, sunitinib, temozolomide, valproic acid, acetaminophen, diclofenac, and indomethacin, together with environmental or experimental chemicals including bisphenol A, atrazine, rotenone, tetrachlorodibenzodioxin, trichloroethylene, and others. The CTD records therefore document diverse chemicals linked to alterations in *FANCE* expression, although these curated associations were derived under differing biological and experimental settings. Genes sharing similar chemical-interaction profiles with *FANCE* were also identified (Table 4). *SNAPC4* had the maximal similarity index (0.3333), followed by *PACSIN3* (0.3273), *FAM110A* (0.3267), *MINDY3* (0.3238), and *DCUN1D3* (0.3235). The number of chemicals shared with *FANCE* ranged from 29 to 39 for the 20 genes listed, with *PAK1IP1* possessing the largest number of common interacting chemicals (*n* = 39). These similarities reflect overlap among CTD-curated chemical interaction records and do not imply direct molecular interactions between *FANCE* and the listed genes.

### 3.10. FANCE-Associated Transcriptional Profile Is Enriched for Cell Cycle, DNA Replication, and DNA Repair Programs in LIHC

**Figure 12:**
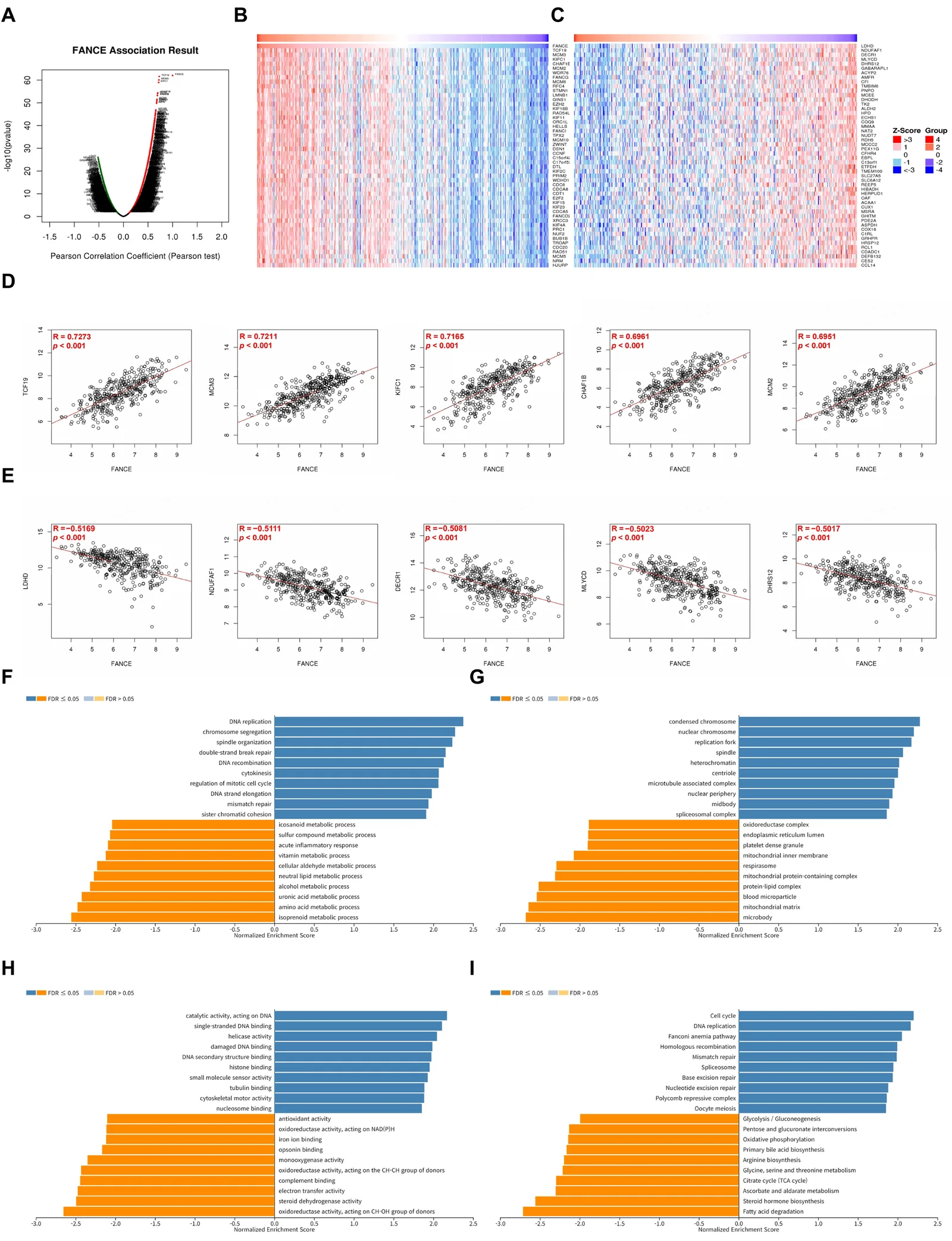
Co-expression and functional enrichment analyses of FANCE in LIHC. (**A**) Genome-wide Pearson correlation profile of genes associated with *FANCE* expression. (**B**,**C**) Heatmaps presenting the top 50 positively and negatively correlated genes. (**D**,**E**) Correlation plots for five genes demonstrating the strongest positive and negative correlations with *FANCE*, respectively. (**F**–**H**) GO enrichment analyses for biological process, cellular component, and molecular function categories. (**I**) KEGG pathway enrichment analysis. Normalized positive and negative enrichment scores indicate enrichment at the corresponding ends of the *FANCE*-correlated gene ranking. FDR ≤ 0.05 was considered statistically significant.

Genome-wide correlation analysis was performed to characterize the transcription profile related to *FANCE* in TCGA–LIHC. Numerous transcripts were significantly positively or negatively correlated with *FANCE* expression (Figure 12A); 50 genes with the most prominent positive and negative correlations are presented in Figure 12B,C. Of the positively correlated genes examined individually, *TCF19* exhibited the highest correlation with *FANCE* (R = 0.7273, *p* < 0.001), followed by *MCM3* (R = 0.7211), *KIFC1* (R = 0.7165), *CHAF1B* (R = 0.6964), and *MCM2* (R = 0.6951) (all *p* < 0.001; Figure 12D). Negative correlations were observed with *LDHD* (R = −0.6596), *NDUFAF1* (R = −0.6511), *DECR1* (R = −0.6501), *MLYCD* (R = −0.6523), and *DHRS12* (R = −0.6617) (all *p* < 0.001; Figure 12E).

GSEA was then applied to the ranked *FANCE*-related transcriptional profile. GO biological process analysis showed positive enrichment primarily for DNA replication, chromosome segregation, cell-cycle regulation, and DNA repair processes, with DNA replication showing the highest enrichment (NES = 2.353, FDR = 0; Figure 12F). Several metabolic processes were enriched in the opposite direction.

GO cellular component analysis similarly highlighted chromosome-, replication fork-, and spindle-related components, with condensed chromosome showing the highest positive enrichment (NES = 2.270, FDR = 0; Figure 12G), whereas several mitochondrial components were negatively enriched.

GO molecular functions analysis was characterized by enrichment of DNA-related catalytic, binding, and helicase activities, with catalytic activity acting on DNA showing the highest positive enrichment (NES = 2.174, FDR = 0; Figure 12H); several oxidoreductase-related functions were negatively enriched.

KEGG analysis further identified positive enrichment of cell-cycle and DNA repair-related pathways (Figure 12I), including cell cycle (NES = 2.193, FDR = 0), DNA replication (NES = 2.147, FDR = 0), FA pathway (NES = 2.056, FDR = 0), and homologous recombination (NES = 2.004, FDR = 0). Metabolic pathways, including oxidative phosphorylation, were negatively enriched. Complete GSEA results are provided in Table S3. Taken together, the *FANCE*-associated transcriptional profile was primarily enriched for cell cycle progression, DNA replication, and multiple DNA repair processes in LIHC, whereas several metabolic pathways were enriched in the opposite direction.

### 3.11. Genes Positively Correlated with FANCE Are Frequently Associated with Higher Survival Risk in LIHC

**Figure 13:**
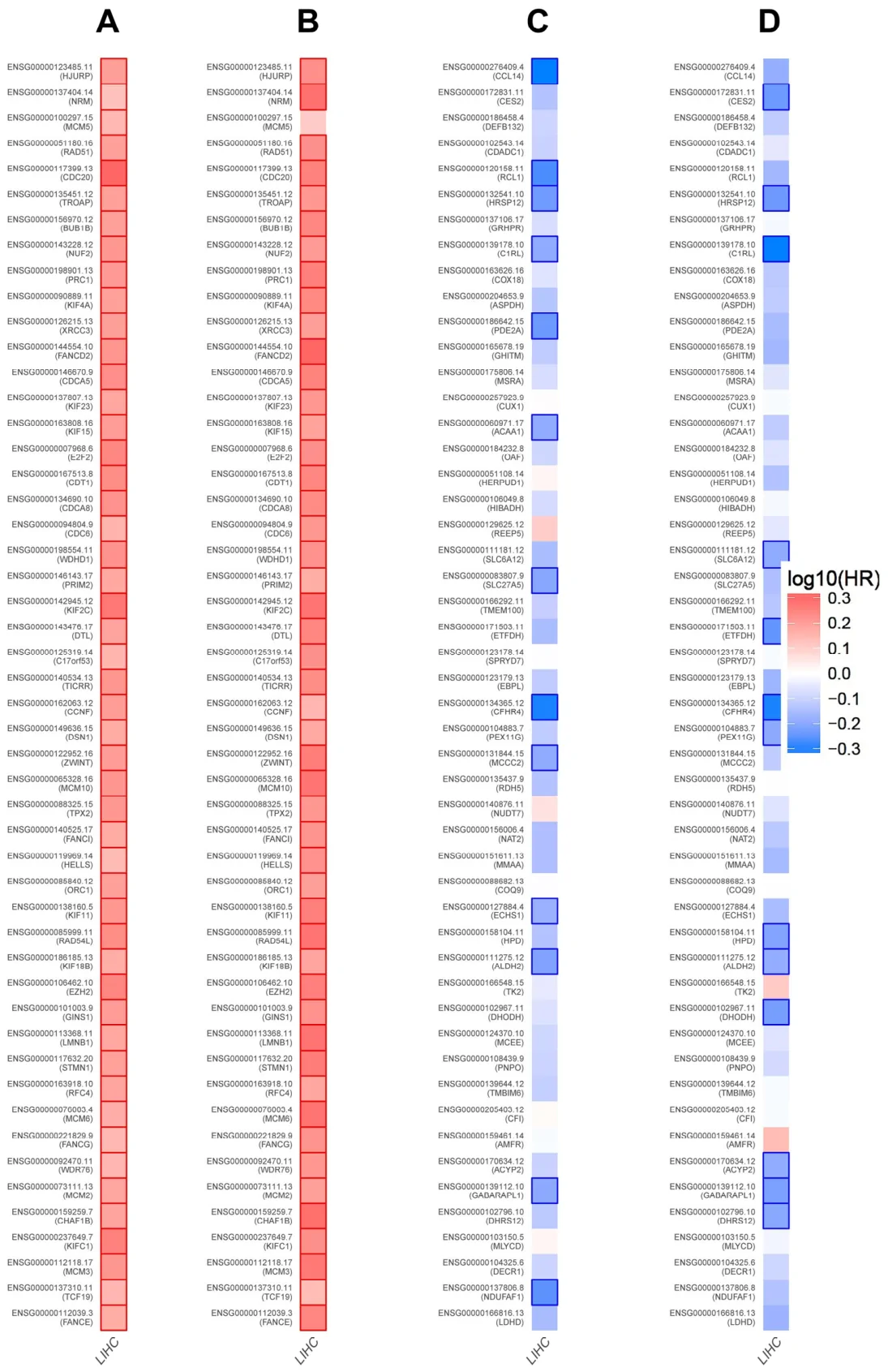
Prognostic analyses of *FANCE*-associated genes in LIHC. Survival analyses of the top 50 genes positively and negatively correlated with *FANCE* expression. (**A**) OS and (**B**) RFS analyses of positively correlated genes. (**C**) OS and (**D**) RFS analyses of negatively associated genes. Color intensity represents the log10(HR), with red and blue indicating increased and reduced hazards, respectively. Outlined boxes denote statistically significant survival relationships (*p* < 0.05).

The prognostic relevance of the *FANCE* co-expression profile was further examined by evaluating OS and RFS for the top 50 positively and negatively correlated genes (Figure 13). All 50 positively correlated genes were significantly linked to increased hazard for OS (Figure 13A). A similar pattern was observed for RFS, with 49 of the 50 genes showing significant positive hazard estimates (Figure 13B).

The negatively correlated gene set exhibited a different survival profile; 13 of the 50 genes were significantly linked to reduced hazard for OS, and 13 demonstrated marked reductions in the hazard for RFS (Figure 13C,D). The remaining genes either did not reach statistical significance or were less consistently associated with survival patterns. Thus, genes exhibiting positive transcriptional relationships with *FANCE* were frequently accompanied by higher survival risk in LIHC, whereas reduced hazard was more commonly detected within the negatively correlated gene set. These survival outcomes at the gene level provide prognostic context for the *FANCE* co-expression profile but do not establish independent prognostic effects for individual genes.

### 3.12. FANCE Methylation Is Related to a Distinct Transcriptional and Prognostic Profile in LIHC

**Figure 14:**
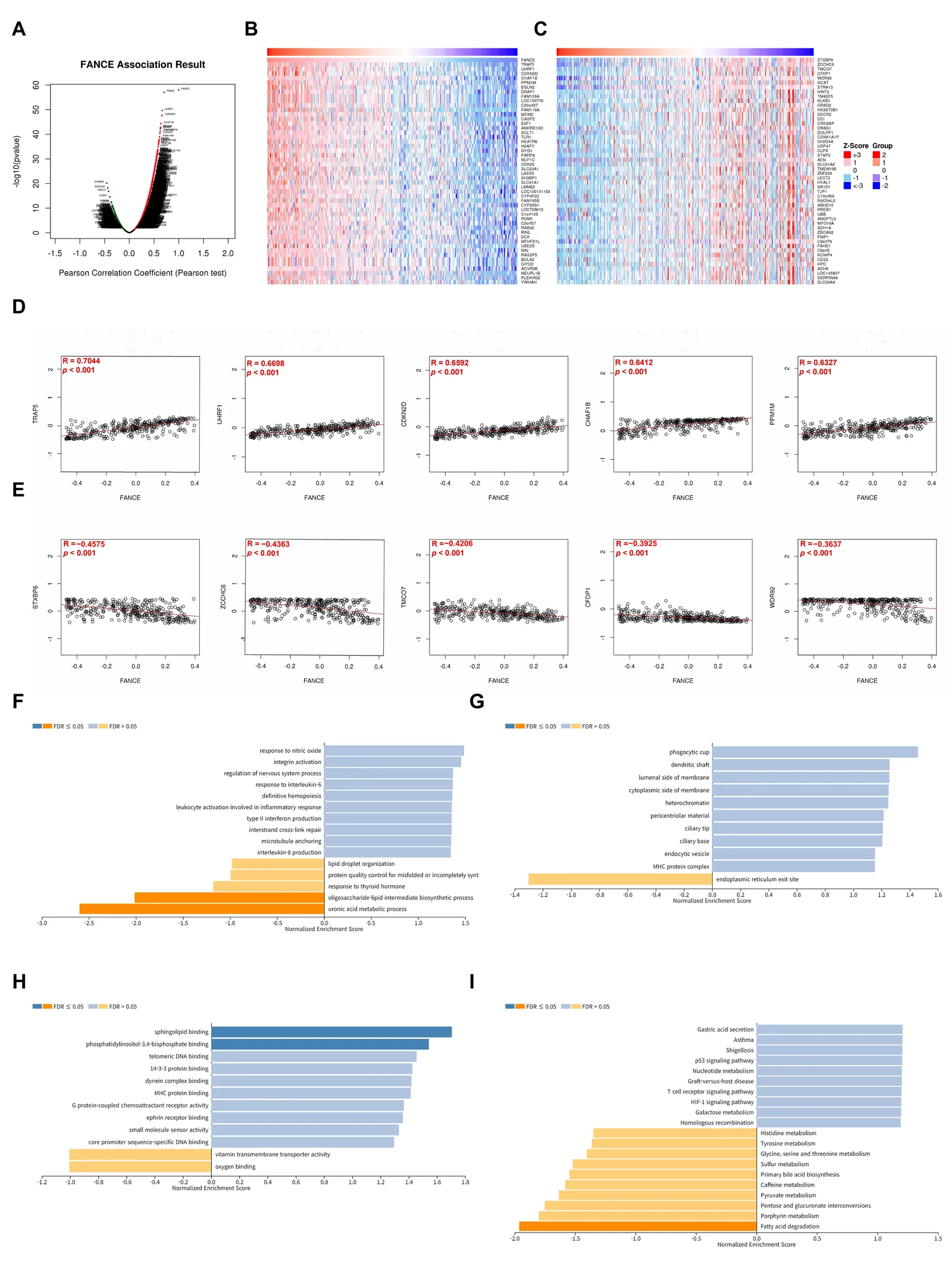
Transcriptomic correlation and functional enrichment analyses associated with *FANCE* methylation in LIHC. (**A**) Genome-wide Pearson correlation profile of genes related to *FANCE* methylation. (**B**,**C**) Heatmaps showing the top 50 positively and negatively correlated genes, respectively. (**D**,**E**) Correlation plots for the five genes exhibiting the strongest positive and negative correlations with *FANCE* methylation, respectively. (**F**–**H**) GO enrichment analyses for biological process, cellular component, and molecular function categories. (**I**) KEGG pathway enrichment analysis. Normalized positive and negative enrichment scores indicate enrichment at the corresponding ends of the *FANCE* methylation-correlated gene ranking. FDR ≤ 0.05 was considered statistically significant.

To determine the transcriptional features related to *FANCE* methylation, genome-wide Pearson correlation analysis was performed in LIHC (Figure 14A–C). Among the genes examined individually, *TRAF5* showed the strongest positive correlation with *FANCE* methylation (R = 0.7044, *p* < 0.001), followed by *UHRF1* (R = 0.6698), *CDKN2D* (R = 0.6592), *CHAF1B* (R = 0.6412), and *PPM1M* (R = 0.6327) (all *p* < 0.001; Figure 14D). *STXBP6* (R = −0.4575), *ZCCHC6* (R = −0.4363), *TMCO7* (R = −0.4206), *CFDP1* (R = −0.3925), and *WDR92* (R = −0.3637) were found to be negatively correlated (all *p* < 0.001; Figure 14E). GSEA was subsequently applied to the methylation-related transcriptional profile. In the GO biological process category, oligosaccharide–lipid intermediate biosynthetic process and uronic acid metabolic process were significantly negatively enriched after FDR correction, whereas the positively ranked terms did not meet the FDR ≤ 0.05 criterion (Figure 14F). None of the GO cellular component terms satisfied the FDR ≤0.05 criterion (Figure 14G). In the GO molecular function category, sphingolipid binding and phosphatidylinositol-3,4-bisphosphate binding demonstrated remarkable positive enrichment (FDR ≤ 0.05; Figure 14H).

**Figure 15:**
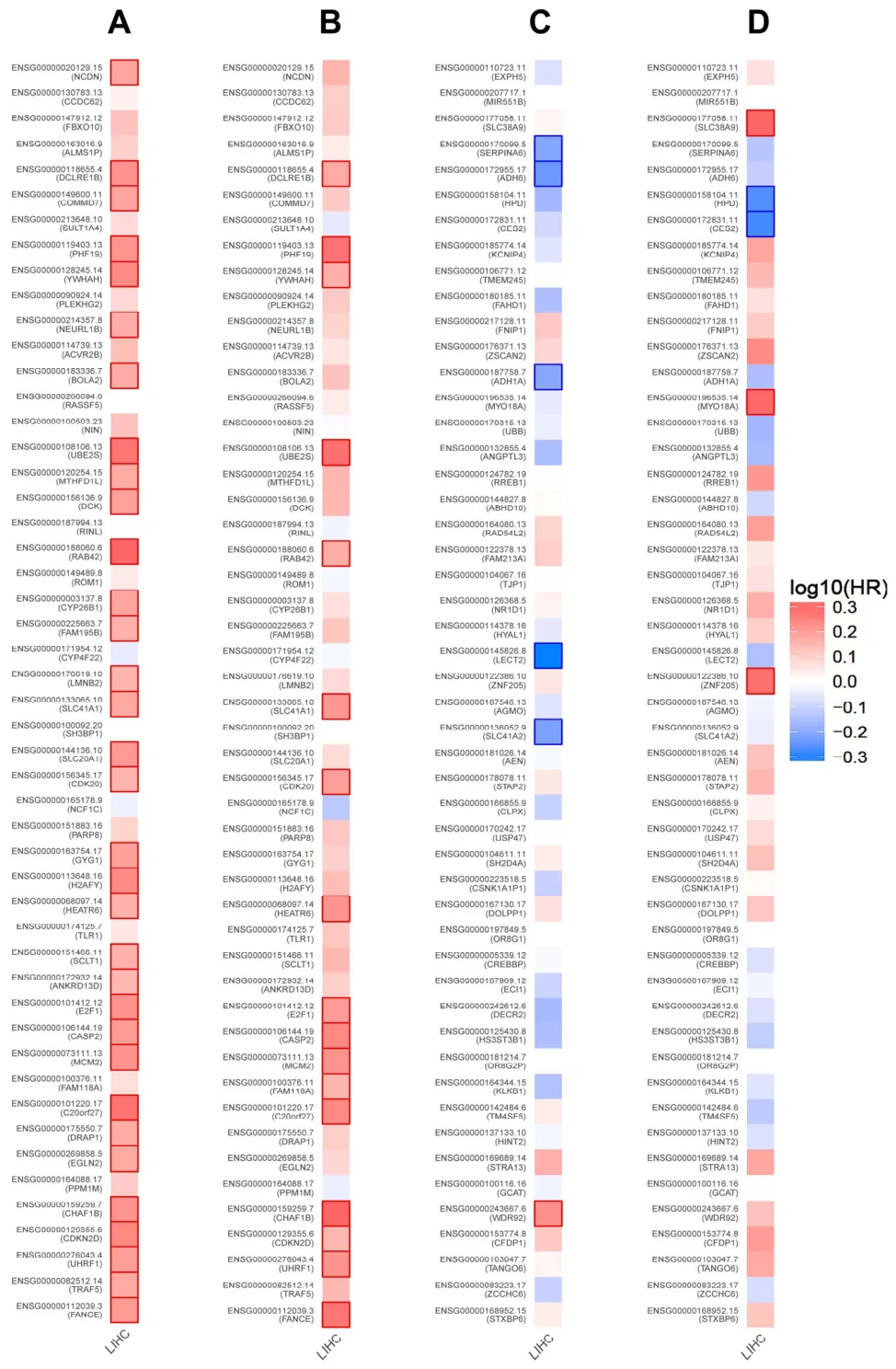
Prognostic analyses of genes associated with *FANCE* methylation in LIHC. OS and RFS analyses, respectively, for the top 50 genes positively (**A**,**B**) and negatively (**C**,**D**) correlated with *FANCE* methylation. Heatmap colors indicate log10-transformed HRs, with red and blue representing HR > 1 and <1, respectively. Outlined boxes indicate statistically significant survival associations (*p* < 0.05).

KEGG analysis revealed significant negative enrichment of fatty acid degradation (FDR ≤ 0.05), whereas the positively ranked pathways did not satisfy the FDR threshold (Figure 14I). Overall, the statistically supported enrichment results were relatively limited and differed from the prominent cell cycle and DNA repair profiles identified in *FANCE* expression-based analysis. The prognostic relevance of the methylation-related gene set was subsequently evaluated (Figure 15). Of the 50 genes positively correlated with *FANCE* methylation, 33 and 17 were significantly linked to increased hazard for OS and RFS, respectively (Figure 15A,B). The negatively correlated gene set showed mixed prognostic directions: for OS, *SERPINA6*, *ADH6*, *ADH1A*, *LECT2*, and *SLC41A2* were associated with reduced hazard, whereas *WDR92* was linked with increased hazard (Figure 15C). For RFS, *HPD* and *CES2* were associated with reduced hazard, whereas *SLC38A9*, *MYO18A*, and *ZNF205* were linked to increased hazard (Figure 15D). These findings indicate that the *FANCE*-methylation-related transcriptional profile contained genes with varying prognostic directions, although elevated survival risk was more frequent within the positively correlated gene set.

### 3.13. The FANCE Protein Interaction Network Is Primarily Linked to FA and DNA-Repair Functions

**Figure 16:**
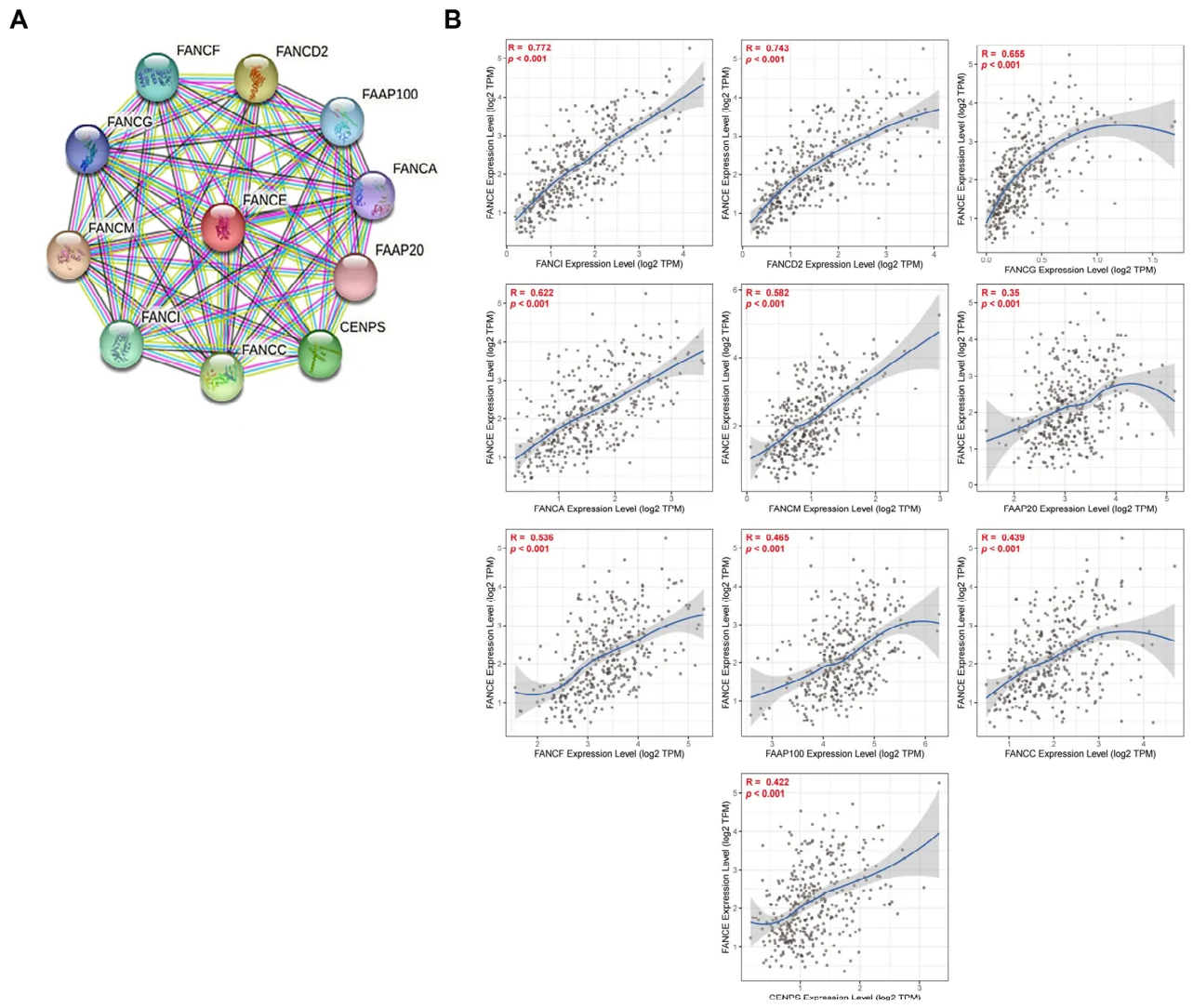

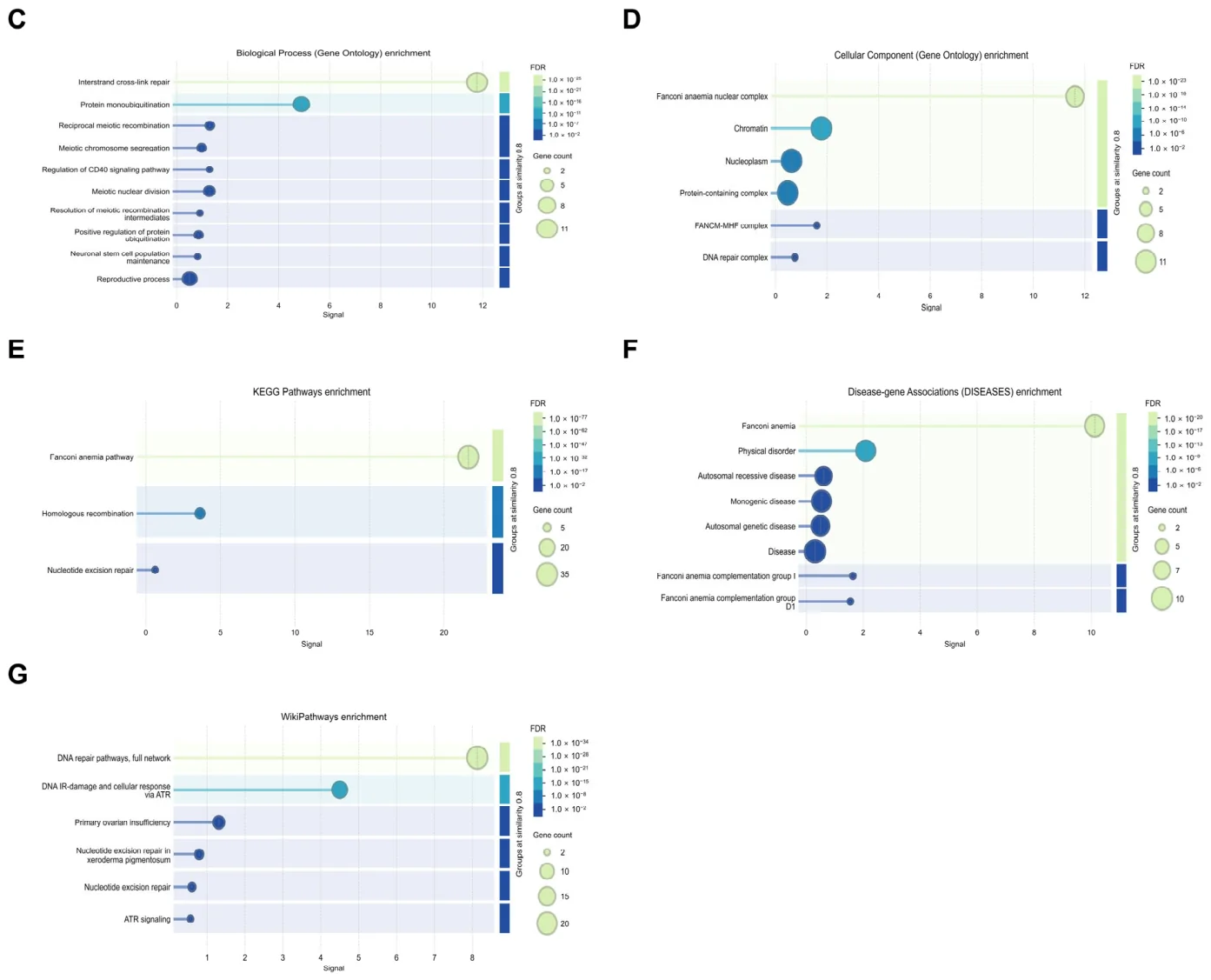
Protein–protein interaction and functional enrichment analyses of *FANCE* in LIHC. (**A**) *FANCE*-centered protein–protein interaction network showing 10 partners identified using STRING. (**B**) Correlations between *FANCE* expression and genes encoding these interaction partners in LIHC. (**C**,**D**) GO enrichment analyses for biological process and cellular component categories, respectively. (**E**) KEGG pathway enrichment analysis. (**F**) Disease–gene association enrichment analysis. (**G**) WikiPathways enrichment analysis. Bubble size indicates the number of genes assigned to each term, and color represents FDR.

A PPI network was constructed to characterize proteins functionally connected to *FANCE* (Figure 16A). The network contained *FANCE*, *FANCF*, *FANCD2*, *FAAP100*, *FANCA*, *FAAP20*, *CENPS*, *FANCC*, *FANCI*, *FANCM*, and *FANCG*, most of which are established components or partners of the FA machinery. The transcriptional relationships between *FANCE* and genes encoding these proteins in LIHC were then examined (Figure 16B). FANCE expression was positively correlated with each of the ten genes (*p* < 0.001 for all). *FANCI* showed the strongest correlation (R = 0.772), followed by *FANCD2* (R = 0.743), *FANCG* (R = 0.665), *FANCA* (R = 0.622), and *FANCM* (R = 0.592). Positive associations were also identified for *FANCF* (R = 0.536), *FAAP100* (R = 0.465), *FANCC* (R = 0.439), *CENPS* (R = 0.422), and *FAAP20* (R = 0.350). Functional enrichment analysis further characterized the biological context of such a network. GO analysis primarily identified interstrand cross-link repair and FA- and DNA repair-related cellular components (Figure 16C,D). KEGG analysis highlighted the FA pathway, homologous recombination, and nucleotide excision repair (Figure 16E), while disease–gene analysis was dominated by FA-related terms (Figure 16F). WikiPathways analysis further identified the DNA repair pathway and ATR-related pathways (Figure 16G). Taken together, the PPI and enrichment findings place *FANCE* within a tightly connected network dominated by FA proteins and DNA repair processes. The concurrent transcriptional correlations in LIHC further support a coordinated expression of *FANCE* with several members of this network, although these database-derived associations do not establish direct functional interactions within LIHC cells.

## 4. Discussion

The present study provides an integrated characterization of *FANCE* in LIHC by linking its expression and prognostic significance with DNA methylation, ncRNA-related networks, transcriptome profiles, protein interactions, treatment-related features, and immune cell infiltration. Although studies have reported increased *FANCE* expression and poor survival in LIHC, the present report extends these observations by integrating multiple molecular and clinical features within a single analytical framework [21,22]. Of particular interest, several analytical approaches highlighted cell cycle regulation, DNA replication, and genome maintenance pathways. Such confluence provides additional biological context for the reported prognostic relevance of *FANCE* rather than simply reproducing its expression and survival patterns.

The transcriptional findings were consistent with the established role of *FANCE* within the FA DNA repair machinery. Genes positively correlated with *FANCE* were enriched in DNA replication, chromosome segregation, cell cycle regulation, homologous recombination, mismatch repair, and the FA pathway. In contrast, genes that were negatively correlated with *FANCE* were preferentially related to oxidative phosphorylation and hepatic metabolic processes. PPI analysis provided a complementary finding, placing *FANCE* in a network containing *FANCI*, *FANCD2*, *FANCA*, *FANCC*, *FANCG*, *FANCM*, and other FA-related proteins, with enrichment primarily involving interstrand cross-link repair and genome maintenance. In conjunction, the concordance between co-expression, survival, pathway, and protein-interaction analyses places higher *FANCE* expression within a transcriptional context characterized by enrichment of cell cycle and DNA repair activity and reduced representation of several metabolic programs. Consistent with this context, when *FANCE* and *MKI67* were included simultaneously in a multivariable Cox model, the association between *FANCE* expression and OS was attenuated and no longer statistically significant, whereas *MKI67* remained significantly associated with OS. These findings do not establish that *FANCE* itself drives these molecular changes, which may also reflect broader biological features of the tumors, including a proliferative phenotype or differences in tumor differentiation; nevertheless, they provide a coherent biological framework within which its increased expression in LIHC can be interpreted.

DNA methylation analysis added an epigenetic dimension to this framework. *FANCE* promoter methylation was lower in LIHC than in normal liver. Methylation patterns were not uniform at each CpG level. Several CpG sites were negatively correlated with *FANCE* expression, whereas cg06209740 was positively correlated, indicating that promoter-level averages alone may not fully describe the *FANCE* methylation landscape. Of particular interest, cg03030757 was negatively correlated with *FANCE* expression, and enhanced methylation at this site was linked to longer survival. Nevertheless, these findings do not demonstrate that methylation at cg03030757 or any other CpG site directly controls *FANCE* transcription. Thus, the present data support a relationship between *FANCE* methylation and its broader molecular context but do not establish a specific epigenetic mechanism that could be responsible for increased *FANCE* expression.

ncRNA analysis identified hsa-miR-101-3p as a candidate particularly relevant for further investigation. This candidate was selected based on the results of this combined analysis rather than on network connectivity alone. The expression of hsa-miR-101-3p was lower in LIHC and negatively correlated with *FANCE* expression, and its higher expression was linked to longer OS. This analytical pattern was also biologically relevant as previous studies have consistently reported reduced miR-101-3p expression and tumor suppressive activity in HCC. In experimental studies, miR-101-3p has been linked to the inhibition of proliferation, migration, invasion, and other tumor-related phenotypes through several molecular targets, including *RAP1B* and *HGF/c-MET* [52,53,54]. More recent evidence has further connected miR-101-3p with the suppression of BIRC5/PI3K–AKT signaling and epithelial–mesenchymal transition in HCC [55]. Therefore, the current findings and previously reported experimental evidence provided a rationale to prioritize hsa-miR-101-3p in subsequent ncRNA analyses. Nevertheless, the negative correlation between hsa-miR-101-3p and *FANCE* does not establish direct targeting and requires experimental confirmation.

The hsa-miR-101-3p-centered analysis identified multiple lncRNAs, circRNA-linked genes, pseudogenes, and sncRNAs. The expression of several candidates was altered in LIHC, showed marked correlations with hsa-miR-101-3p or *FANCE*, and exhibited survival-related findings. Previous work identified a GSEC/miR-101-3p/SNX16/PAPOLG axis in HCC wherein increased GSEC and reduced miR-101-3p were linked to shorter survival [56]. However, these findings require careful interpretation as the analyses were based on predicted interactions and transcript-level correlations, and circRNA-linked analysis relied on host- or circRNA-linked gene expression rather than direct measurements of circRNA isoforms. These gene-level patterns may also partly reflect the broader proliferative transcriptional profile associated with *FANCE*, rather than circRNA-specific regulation. Therefore, the results identify potential candidate components for subsequent investigation but do not establish direct *FANCE*–miRNA targeting, competing endogenous RNA activity, or other specific regulatory relationships.

Immune analyses further indicated that variations in *FANCE* expression coincided with differences in inferred immune cell composition in LIHC. CD8+ T-cell estimates were generally positively correlated with *FANCE* expression, whereas the direction of macrophage-related estimates differed according to the deconvolution algorithm. Consequently, immune findings are better interpreted as evidence that *FANCE* expression occurs within an immunologically heterogeneous context rather than as indicative of a uniformly immune-active or immune-suppressed tumor state. As these estimates were derived from bulk transcriptomic datasets, they cannot determine immune cell functional states, spatial localization, or direct interactions between *FANCE*-expressing tumor cells and immune populations.

Treatment-related analyses provide another exploratory aspect of the present study. Elevated *FANCE* expression was also detected in responders to nivolumab, pembrolizumab, and ipilimumab, although the ROC values indicated only modest discrimination. However, treatment cohorts varied in cancer type, therapeutic setting, and clinical characteristics, were not matched for potential clinical confounders, and formal analyses of expression-by-treatment interactions were not included. Thus, the present findings must not be interpreted as evidence that *FANCE* or hsa-miR-101-3p predicts therapeutic benefit in LIHC. Rather, they identify treatment-related patterns that warrant testing in appropriately designed LIHC cohorts.

A notable feature of this analysis is the identification of related biological themes through several analytical approaches. The most consistent findings were increased *FANCE* expression in LIHC, its association with shorter survival, and a *FANCE*-associated transcriptional profile characterized by cell cycle, DNA replication, and DNA repair processes. Methylation, ncRNA, immune, PPI, and treatment-related analyses provided additional molecular and clinical contexts. The principal contribution therefore lies not in establishing a new *FANCE*-dependent mechanism but in extending earlier expression- and prognosis-based observations by characterizing its broader molecular context and identifying specific regulatory candidates and biological processes for subsequent experimental investigation. Such a distinction is crucial because agreement of cross-platform computational results may support biological plausibility but cannot be regarded as independent validation or substitute for mechanistic validation.

Several limitations should be considered. First, the study was retrospective and relied primarily on publicly available datasets. Some analytical platforms use overlapping TCGA–LIHC samples, and concordant results obtained from them cannot always be regarded as independent validation. Moreover, the prognostic and molecular findings were not validated in an independent LIHC cohort, which limits their generalizability beyond the datasets analyzed in this study. Second, although multivariable Cox analysis was performed with adjustment for age, gender, and tumor stage, other potentially relevant prognostic factors, including tumor grade, vascular invasion, TP53 status, and AFP level, were not available for inclusion in the TIMER3 Gene_Outcome model. Third, protein expression was evaluated based on HPA immunohistochemical images without protein quantification or independent tissue validation. Fourth, numerous subgroup, CpG, immune cell, ncRNA, and pathway analyses employed platform-specific statistical procedures, increasing the possibility of false-positive findings despite the use of significance thresholds and FDR correction where available. Fifth, immune cell estimates were derived from bulk transcriptomic deconvolution and therefore lack cell-level and spatial resolution. Sixth, the proposed miRNA- and ncRNA-related relationships were derived from prediction, expression, and correlation analyses and not experimentally verified. Seventh, the treatment-response analyses employed heterogeneous external cohorts and cannot establish LIHC-specific predictive utility. Finally, no experiments were performed to determine whether altering *FANCE* expression or methylation directly affects the molecular phenotypes identified here.

These limitations also define several priorities for subsequent investigations. Targeted methylation analyses may determine whether CpG sites, such as cg03030757, contribute to *FANCE* transcriptional regulation. The proposed relationship between hsa-miR-101-3p and *FANCE* should be examined using miRNA gain- and loss-of-function approaches together with direct target and rescue experiments. Independent clinical cohorts are also necessary to determine whether the prognostic and molecular findings are reproducible beyond TCGA-LIHC and whether *FANCE* provides prognostic information beyond established clinical variables. Finally, single-cell and spatial approaches could clarify immune cell patterns identified from bulk transcriptomic data.

## 5. Conclusions

In summary, the present integrative bioinformatic analysis extends the established observations of increased *FANCE* expression and poor prognosis in LIHC by defining a broader molecular context involving DNA methylation, an hsa-miR-101-3p-centered ncRNA network, immune cell infiltration, treatment-related features, and coordinated transcriptional and protein-interaction networks. The most consistent finding was the association of the *FANCE*-related transcriptional profile with cell cycle, DNA replication, FA, and other genome-maintenance-associated processes, accompanied by survival-related patterns in *FANCE* and many positively correlated genes. Additionally, hsa-miR-101-3p was identified as a candidate of particular interest based on its reduced expression in LIHC, negative correlation with *FANCE*, survival relationship, and earlier reported tumor-suppressive functions in HCC. These findings provide a molecular framework for prioritizing *FANCE* and selected regulatory candidates for further investigation rather than confirming direct regulatory mechanisms or clinical utility. Experimental validation and independent clinical studies are required to determine the biological role of *FANCE* and to assess whether it can provide clinically meaningful prognostic or therapeutic information in LIHC.

## Figures and Tables

**Table 1:** Correlations between *FANCE* expression patterns and immune infiltration estimates in LIHC across multiple deconvolution algorithms.

Gene	Algorithm	Cell Type	R	*p*-Value	Algorithm	Cell Type	R	*p*-Value
*FANCE*	XCELL	Class-switched memory B cell	0.151	<0.01	CONSENSUS_TME	B cell	0.277	<0.001
		B cell	0.273	<0.001		Myeloid dendritic cells	0.109	<0.05
		B-cell memory	0.107	<0.05		Eosinophil	0.212	<0.001
		Common lymphoid progenitor	0.433	<0.001		MAST cell_CONSENSUS	0.17	<0.01
		Myeloid dendritic cell activated	0.158	<0.01		Monocyte	0.176	<0.01
		Myeloid dendritic cells	0.139	<0.01		NK cell	0.115	<0.05
		Plasmacytoid dendritic cells	−0.121	<0.05		T-cell CD4+	0.27	<0.001
		Endothelial cell	−0.443	<0.001		T-cell CD8+	0.165	<0.01
		Granulocyte-monocyte progenitor	−0.276	<0.001		T-cell gamma delta	0.453	<0.001
		Hematopoietic stem cells	−0.372	<0.001		T cell regulatory (Tregs)	0.189	<0.001
		Macrophage M2	−0.391	<0.001	CIBERSORT-ABS	B-cell plasma	0.234	<0.001
		Macrophage	−0.182	<0.001		B-cell memory	0.114	<0.05
		Mast cell	0.273	<0.001		Myeloid dendritic cells were resting	0.162	<0.01
		Monocyte	0.152	<0.01		Macrophage M0	0.261	<0.001
		NK cell	0.127	<0.05		Macrophage M1	0.294	<0.001
		T-cell NK	0.182	<0.001		Macrophage M2	0.35	<0.001
		T-cell CD4+ central memory	−0.164	<0.01		NK cell activated	0.35	<0.001
		T-cell CD4+ effector memory	−0.137	<0.05		NK cell resting	−0.129	<0.05
		T-cell CD4+ memory	0.175	<0.01		Neutrophil	0.12	<0.05
		T-cell CD4+ Th1	0.155	<0.01		T-cell CD4+ memory activation	0.152	<0.01
		T-cell CD4+ Th2	0.502	<0.001		T-cell CD4+ memory resting	0.179	<0.001
	TIMER	B cell	0.325	<0.001		NK cell activated	0.35	<0.001
		Myeloid dendritic cells	0.489	<0.001		T-cell CD8+	0.274	<0.001
		Macrophage	0.388	<0.001		T cell follicular helper	0.398	<0.001
		Neutrophil	0.222	<0.001		T cell regulatory (Tregs)	0.374	<0.001
		T-cell CD4+	0.241	<0.001	CIBERSORT	B-cell naïve	−0.139	<0.01
		T-cell CD8+	0.15	<0.01		B-cell plasma	0.14	<0.01
	TIDE	CD8	0.351	<0.001		Myeloid dendritic cells were resting	0.115	<0.05
		CTL	0.279	<0.001		Macrophage M0	0.154	<0.01
		MSI Score	−0.132	<0.05		Myeloid dendritic cells were resting	0.115	<0.05
		*CD274*	0.309	<0.001		Macrophage M2	−0.122	<0.05
		Dysfunction_TIDE	−0.346	<0.001		Monocyte	−0.205	<0.001
		Exclusion	0.348	<0.001		NK cell resting	−0.147	<0.01
		MDSC	0.35	<0.001		T-cell CD4+ memory activation	0.15	<0.01
		*IFNG*	0.298	<0.001		T cell follicular helper	0.211	<0.001
	ImmuCellAI	MAIT	0.247	<0.001		T cell regulatory (Tregs)	0.181	<0.001
		Macrophage	0.206	<0.001	QUANTISEQ	B cell	0.421	<0.001
		NKT	−0.169	<0.01		Macrophage M1	0.249	<0.001
		CD4_naïve	0.122	<0.05		Macrophage M2	0.263	<0.001
		CD4_T	0.107	<0.05		Monocyte	0.183	<0.001
		Th1	−0.117	<0.05		NK cell	−0.111	<0.05
		Tr1	0.248	<0.001		T-cell CD4+ (non-regulatory)	0.153	<0.01
		CD8_T	0.306	<0.001		T-cell CD8+	0.363	<0.001
		Cytotoxic	0.112	<0.05		T cell regulatory (Tregs)	0.345	<0.001
		Exhausted	0.228	<0.001	ABIS	B-cell naïve	−0.223	<0.001
		Gamma_delta	−0.242	<0.001		B cell plasma immature	0.225	<0.001
	EPIC	Cancer-associated fibroblasts	0.266	<0.001		B-cell memory	0.405	<0.001
		Macrophage	−0.476	<0.001		Basophil	0.522	<0.001
		NK cell	−0.113	<0.05		Myeloid dendritic cells	0.409	<0.001
	MCPCOUNTER	B cell	0.35	<0.001		Plasmacytoid dendritic cells	0.525	<0.001
		Cancer-associated fibroblasts	0.198	<0.001		Monocyte conventional	0.175	<0.01
		Myeloid dendritic cells	0.371	<0.001		Monocyte non-conventional	0.188	<0.001
		Macrophage/Monocyte	0.404	<0.001		T-cell CD4+ naïve	0.409	<0.001
		Monocyte	0.404	<0.001		T-cell CD8+ memory	0.237	<0.001
		NK cell	0.247	<0.001		T-cell CD8+ naïve	−0.123	<0.05
		T-cell CD8+	0.325	<0.001				

Footnote: Nominal *p*-values were not adjusted for multiple comparisons; therefore, these findings should be interpreted as exploratory.

**Table 2:** Associations between *FANCE*-related immune infiltration estimates and survival outcomes in LIHC across multiple deconvolution algorithms.

Gene	Algorithm	Cell Type	OS		PFI		PFS		DSS		DFI	
			Z	*p*-Value	Z	*p*-Value	Z	*p*-Value	Z	*p*-Value	Z	*p*-Value
*FANCE*	XCELL	B-cell plasma	−2.112	<0.05	−2.324	<0.05	−2.23	<0.05	−2.102	<0.05	−2.027	<0.05
		T-cell CD4+ Th2	2.691	<0.01	1.862	0.063	1.748	0.08	2.701	<0.01	1.712	0.087
		T-cell CD8+ central memory	−1.998	<0.05	−2.463	<0.05	−2.245	<0.05	−1.988	<0.05	−1.978	<0.05
	TIMER	Macrophage	2.332	<0.05	1.772	0.076	1.926	0.054	2.335	<0.05	1.814	0.07
	TIDE	CTL.flag	−2.34	<0.05	−3.354	<0.01	−3.363	<0.001	−2.343	<0.05	−3.461	<0.01
	ImmuCellAI	Macrophage	2.381	<0.05	2	<0.05	2.164	<0.05	2.395	<0.05	1.284	0.199
		Monocyte	−2.069	<0.05	−1.959	0.05	−1.675	0.094	−2.091	<0.05	−2.095	<0.05
		Th1	2.392	<0.05	2.326	<0.05	2.107	<0.05	2.409	<0.05	2.215	<0.05
		Cytotoxic	−2.287	<0.05	−1.988	<0.05	−2.106	<0.05	−2.28	<0.05	−1.117	0.264
		Gamma_delta	−3.348	<0.01	−3.109	<0.01	−3.22	<0.01	−3.357	<0.01	−2.674	<0.01
	EPIC	Macrophage	−2.229	<0.05	−2.048	<0.05	−2.249	<0.05	−2.244	<0.05	−1.489	0.136
	CONSENSUS_TME	NK cell	−2.019	<0.05	−2.785	<0.01	−2.548	<0.05	−2.021	<0.05	−2.589	<0.05
		T-cell CD8+	−2.145	<0.05	−3.01	<0.01	−2.776	<0.01	−2.14	<0.05	−2.842	<0.01
		T cell regulatory (Tregs)	−2.12	<0.05	−2.98	<0.01	−2.751	<0.01	−2.112	<0.05	−2.944	<0.01
	CIBERSORT-ABS	T-cell CD4+ memory resting	−2.231	<0.05	−2.298	<0.05	−1.957	0.05	−2.225	<0.05	−2.537	<0.05
		T-cell CD8+	−2.109	<0.05	−2.626	<0.01	−2.375	<0.05	−2.108	<0.05	−2.304	<0.05
	CIBERSORT	Macrophage M0	3.786	<0.001	3.188	<0.01	3.079	<0.01	3.797	<0.001	2.852	<0.01
		Macrophage M2	2.854	<0.01	3.132	<0.01	3.192	<0.01	2.843	<0.01	2.814	<0.01
		T-cell CD4+ memory resting	−2.202	<0.05	−1.989	<0.05	−1.912	0.056	−2.195	<0.05	−2.376	<0.05
		T-cell CD8+	−2.826	<0.01	−3.151	<0.01	−3.164	<0.01	−2.831	<0.01	−2.731	<0.01

Abbreviations: OS, overall survival; PFI, progression-free interval; PFS, progression-free survival; DSS, disease-specific survival; DFI, disease-free interval; Z, Cox regression Z-score. Positive and negative Z-scores indicate unfavorable and favorable survival associations, respectively. Nominal *p*-values were not adjusted for multiple comparisons; therefore, these findings should be interpreted as exploratory.

**Table 3:** CTD-curated chemical compounds reported to increase *FANCE* expression.

Chemical Name	Chemical ID	Interaction Actions
2,3,7,8-Tetrachlorodibenzofuran	C014211	Increases expression
4-Dichlorobenzene	C018511	Increases expression
Acetaminophen	D000082	Increases expression
Atrazine	D001280	Increases expression
Bisphenol A	C006780	Increases expression
Cyclosporine	D016572	Increases expression
Diclofenac	D004008	Increases expression
Dietary fats	D004041	Increases expression
Ethyl methanesulfonate	D005020	Increases expression
Hexabromocyclododecane	C089796	Increases expression
Indomethacin	D007213	Increases expression
Methidathion	C005828	Increases expression
Rotenone	D012402	Increases expression
Sunitinib	D000077210	Increases expression
Temozolomide	D000077204	Increases expression
Tetrachlorodibenzodioxin	D013749	Increases expression
Trichloroethylene	D014241	Increases expression
Tris(1,3-dichloro-2-propyl)phosphate	C016805	Increases expression
Valproic acid	D014635	Increases expression
Vinclozolin	C025643	Increases expression

**Table 4:** Genes with CTD chemical interaction profiles similar to that of *FANCE*.

Gene	Similarity Index	Common Interacting Chemicals
*SNAPC4*	0.3333	32
*PACSIN3*	0.3273	36
*FAM110A*	0.3267	33
*MINDY3*	0.3238	34
*DCUN1D3*	0.3235	33
*TYSND1*	0.3217	37
*NAGPA*	0.3196	31
*TSPAN9*	0.3113	33
*LRRC20*	0.3103	36
*LRRC45*	0.31	31
*TMEM134*	0.31	31
*ANKRD16*	0.3061	30
*COX16*	0.3061	30
*POC1A*	0.3051	36
*RAP1GAP2*	0.3051	36
*PAQR4*	0.3036	34
*PAK1IP1*	0.3023	39
*CNPY4*	0.3021	29
*DIS3L2*	0.301	31
*FAM53C*	0.3	30

## Data Availability

All data are available upon reasonable request from the corresponding author.

## Supplementary Materials

The following supporting information can be downloaded at: https://www.mdpi.com/article/10.3390/medicina62101988/s1, Table S1. Prognostic value of FANCE expression across cancer cohorts. Table S2. Complete PrognoScan survival-analysis results for FANCE across additional cancers cohorts. Table S3. Complete GSEA results for the FANCE-associated transcriptional profile in LIHC, including GO Biological Process, GO Cellular Component, GO Molecular Function, and KEGG pathway analyses. Figure S1. Association between FANCE expression and immune cell infiltration in various cancer types. Heatmap presenting the Spearman correlation coefficients for FANCE expression and the indicated immune cell populations. Color intensity reflects the direction and magnitude of the correlation. * *p* ≤ 0.05 and # FDR ≤ 0.05.

## Abbreviations

The following abbreviations are used in this manuscript:

CI	Confidence interval
circRNA	Circular RNA
DSS	Disease-specific survival
DFI	Disease-free survival
FA	Fanconi anemia
FANCE	Fanconi anemia complementation group E
GO	Gene Ontology
HRs	Hazard ratios
LIHC	Liver hepatocellular carcinoma
lncRNA	Long non-coding RNA
miRNA	Micro RNA
ncRNA	Non-coding RNA
OS	Overall survival
PFS	Progression-free survival
RFS	Recurrence-free survival

## References

[1] Sung H., Ferlay J., Siegel R.L., Laversanne M., Soerjomataram I., Jemal A., Bray F. (2021). Global Cancer Statistics 2020: GLOBOCAN Estimates of Incidence and Mortality Worldwide for 36 Cancers in 185 Countries. CA Cancer J. Clin..

[2] Villanueva A. (2019). Hepatocellular carcinoma. N. Engl. J. Med..

[3] Forner A., Reig M., Bruix J. (2018). Hepatocellular carcinoma. Lancet.

[4] Craig A.J., von Felden J., Garcia-Lezana T. (2020). Tumour evolution in hepatocellular carcinoma. Nat. Rev. Gastroenterol. Hepatol..

[5] Singal A.G., Kanwal F., Llovet J.M. (2023). Global trends in hepatocellular carcinoma epidemiology: Implications for screening, prevention and therapy. Nat. Rev. Clin. Oncol..

[6] Llovet J.M., Kelley R.K., Villanueva A. (2021). Hepatocellular carcinoma. Nat. Rev. Dis. Primers.

[7] Esteller M., Dawson M.A., Kadoch C., Rassool F.V., Jones P.A., Baylin S.B. (2024). The epigenetic hallmarks of cancer. Cancer Discov..

[8] Cai B., Lv D., Qiu Q., Xiong W., Tang H., Bai Y., Zhou S., Hu Y., Safadi R., Wang C. (2026). Epigenetic Alterations in Hepatocellular Carcinoma: Mechanisms and Biomarkers for Precision Therapy. Cancers.

[9] Hung J.-H., Teng C.-F., Hung H.-c., Chen Y.-L., Chen P.-J., Ho C.-L., Chuang C.-H., Huang W. (2024). Genomic instabilities in hepatocellular carcinoma: Biomarkers and application in immunotherapies. Ann. Hepatol..

[10] Li Y., Yu Y., Yang L., Wang R. (2023). Insights into the role of oxidative stress in hepatocellular carcinoma development. Front. Biosci.-Landmark.

[11] Bayard Q., Cordier P., Péneau C., Imbeaud S., Hirsch T.Z., Renault V., Nault J.-C., Blanc J.-F., Calderaro J., Desdouets C. (2022). Structure, dynamics, and impact of replication stress–induced structural variants in hepatocellular carcinoma. Cancer Res..

[12] Naim V. (2024). Understanding DNA replication and replication stress as avenues to combat cancer. Commun. Biol..

[13] Zhang Z., Li H., Chen L., Lu T., Shentu X., Liu Y., Tang Z., Ji S., Qiu Z., Zou Y. (2026). Molecular insights into early malignant transition of hepatocellular carcinoma. Cancer Cell.

[14] Xun Z., Wang Y., Long J., Li Y., Yang X., Sun H., Zhao H. (2023). Development and validation of a genomic instability-related lncRNA prognostic model for hepatocellular carcinoma. Front. Genet..

[15] Peake J.D., Noguchi E. (2022). Fanconi anemia: Current insights regarding epidemiology, cancer, and DNA repair. Hum. Genet..

[16] Ma R., Xu X. (2024). Deciphering the role of post-translational modifications in fanconi anemia proteins and their influence on tumorigenesis. Cancer Gene Ther..

[17] Alcón P., Kaczmarczyk A.P., Ray K.K., Liolios T., Guilbaud G., Sijacki T., Shen Y., McLaughlin S.H., Sale J.E., Knipscheer P. (2024). FANCD2–FANCI surveys DNA and recognizes double-to single-stranded junctions. Nature.

[18] Pace P., Johnson M., Tan W.M., Mosedale G., Sng C., Hoatlin M., De Winter J., Joenje H., Gergely F., Patel K. (2002). *FANCE*: The link between Fanconi anaemia complex assembly and activity. EMBO J..

[19] Taniguchi T., D’Andrea A.D. (2002). The Fanconi anemia protein, *FANCE*, promotes the nuclear accumulation of FANCC. Blood J. Am. Soc. Hematol..

[20] Lemonidis K., Arkinson C., Rennie M.L., Walden H. (2022). Mechanism, specificity, and function of FANCD2-FANCI ubiquitination and deubiquitination. FEBS J..

[21] Takahashi J., Masuda T., Kitagawa A., Tobo T., Nakano Y., Abe T., Ando Y., Kosai K., Kobayashi Y., Matsumoto Y. (2022). Fanconi anemia complementation group E, a DNA repair-related gene, is a potential marker of poor prognosis in hepatocellular carcinoma. Oncology.

[22] Zhou Z., Yin H., Suye S., He J., Fu C. (2023). Pan-cancer analysis of the prognostic and immunological role of Fanconi anemia complementation group E. Front. Genet..

[23] Su L., Bu J., Yu J., Jin M., Meng G., Zhu X. (2024). Comprehensive review and updated analysis of DNA methylation in hepatocellular carcinoma: From basic research to clinical application. Clin. Transl. Med..

[24] Shi Q., Chu Q., Zeng Y., Yuan X., Wang J., Zhang Y., Xue C., Li L. (2023). Non-coding RNA methylation modifications in hepatocellular carcinoma: Interactions and potential implications. Cell Commun. Signal..

[25] Fu S., Debes J.D., Boonstra A. (2023). DNA methylation markers in the detection of hepatocellular carcinoma. Eur. J. Cancer.

[26] Moldogazieva N.T., Zavadskiy S.P., Astakhov D.V., Sologova S.S., Margaryan A.G., Safrygina A.A., Smolyarchuk E.A. (2023). Differentially expressed non-coding RNAs and their regulatory networks in liver cancer. Heliyon.

[27] Shi T., Morishita A., Kobara H., Masaki T. (2021). The role of long non-coding RNA and microRNA networks in hepatocellular carcinoma and its tumor microenvironment. Int. J. Mol. Sci..

[28] Lou W., Ding B., Fu P. (2020). Pseudogene-derived lncRNAs and their miRNA sponging mechanism in human cancer. Front. Cell Dev. Biol..

[29] Xu G., Xu W.-Y., Xiao Y., Jin B., Du S.-D., Mao Y.-l., Zhang Z.-T. (2020). The emerging roles of non-coding competing endogenous RNA in hepatocellular carcinoma. Cancer Cell Int..

[30] Chan J.J., Tay Y. (2018). Noncoding RNA: RNA regulatory networks in cancer. Int. J. Mol. Sci..

[31] Shen K.-Y., Zhu Y., Xie S.-Z., Qin L.-X. (2024). Immunosuppressive tumor microenvironment and immunotherapy of hepatocellular carcinoma: Current status and prospectives. J. Hematol. Oncol..

[32] Gao X., Zuo S. (2023). Immune landscape and immunotherapy of hepatocellular carcinoma: Focus on innate and adaptive immune cells. Clin. Exp. Med..

[33] Cui H., Zhao G., Lu Y., Zuo S., Duan D., Luo X., Zhao H., Li J., Zeng Z., Chen Q. (2025). TIMER3: An enhanced resource for tumor immune analysis. Nucleic Acids Res..

[34] Tang G., Cho M., Wang X. (2022). OncoDB: An interactive online database for analysis of gene expression and viral infection in cancer. Nucleic Acids Res..

[35] Chandrashekar D.S., Bashel B., Balasubramanya S.A.H., Creighton C.J., Ponce-Rodriguez I., Chakravarthi B.V., Varambally S. (2017). UALCAN: A portal for facilitating tumor subgroup gene expression and survival analyses. Neoplasia.

[36] Chandrashekar D.S., Karthikeyan S.K., Korla P.K., Patel H., Shovon A.R., Athar M., Netto G.J., Qin Z.S., Kumar S., Manne U. (2022). UALCAN: An update to the integrated cancer data analysis platform. Neoplasia.

[37] Uhlén M., Fagerberg L., Hallström B.M., Lindskog C., Oksvold P., Mardinoglu A., Sivertsson Å., Kampf C., Sjöstedt E., Asplund A. (2015). Tissue-based map of the human proteome. Science.

[38] Menyhárt O., Nagy Á., Győrffy B. (2018). Determining consistent prognostic biomarkers of overall survival and vascular invasion in hepatocellular carcinoma. R. Soc. Open Sci..

[39] Liu C.-J., Hu F.-F., Xie G.-Y., Miao Y.-R., Li X.-W., Zeng Y., Guo A.-Y. (2023). GSCA: An integrated platform for gene set cancer analysis at genomic, pharmacogenomic and immunogenomic levels. Brief. Bioinform..

[40] Mizuno H., Kitada K., Nakai K., Sarai A. (2009). PrognoScan: A new database for meta-analysis of the prognostic value of genes. BMC Med. Genom..

[41] Koch A., Jeschke J., Van Criekinge W., van Engeland M., De Meyer T. (2019). MEXPRESS update 2019. Nucleic Acids Res..

[42] Modhukur V., Iljasenko T., Metsalu T., Lokk K., Laisk-Podar T., Vilo J. (2018). MethSurv: A web tool to perform multivariable survival analysis using DNA methylation data. Epigenomics.

[43] Chang L., Zhou G., Soufan O., Xia J. (2020). miRNet 2.0: Network-based visual analytics for miRNA functional analysis and systems biology. Nucleic Acids Res..

[44] Li J.-H., Liu S., Zhou H., Qu L.-H., Yang J.-H. (2014). starBase v2.0: Decoding miRNA-ceRNA, miRNA-ncRNA and protein–RNA interaction networks from large-scale CLIP-Seq data. Nucleic Acids Res..

[45] Kang Y.-J., Pan L., Liu Y., Rong Z., Liu J., Liu F. (2025). GEPIA3: Enhanced drug sensitivity and interaction network analysis for cancer research. Nucleic Acids Res..

[46] Kovács S.A., Fekete J.T., Győrffy B. (2023). Predictive biomarkers of immunotherapy response with pharmacological applications in solid tumors. Acta Pharmacol. Sin..

[47] Davis A.P., Wiegers T.C., Sciaky D., Barkalow F., Strong M., Wyatt B., Wiegers J., McMorran R., Abrar S., Mattingly C.J. (2025). Comparative toxicogenomics database’s 20th anniversary: Update 2025. Nucleic Acids Res..

[48] Vasaikar S.V., Straub P., Wang J., Zhang B. (2018). LinkedOmics: Analyzing multi-omics data within and across 32 cancer types. Nucleic Acids Res..

[49] Subramanian A., Tamayo P., Mootha V.K., Mukherjee S., Ebert B.L., Gillette M.A., Paulovich A., Pomeroy S.L., Golub T.R., Lander E.S. (2005). Gene set enrichment analysis: A knowledge-based approach for interpreting genome-wide expression profiles. Proc. Natl. Acad. Sci. USA.

[50] Tang Z., Kang B., Li C., Chen T., Zhang Z. (2019). GEPIA2: An enhanced web server for large-scale expression profiling and interactive analysis. Nucleic Acids Res..

[51] Szklarczyk D., Nastou K., Koutrouli M., Kirsch R., Mehryary F., Hachilif R., Hu D., Peluso M.E., Huang Q., Fang T. (2025). The STRING database in 2025: Protein networks with directionality of regulation. Nucleic Acids Res..

[52] Sheng Y., Ding S., Chen K., Chen J., Wang S., Zou C., Zhang J., Cao Y., Huang A., Tang H. (2014). Functional analysis of miR-101-3p and Rap1b involved in hepatitis B virus-related hepatocellular carcinoma pathogenesis. Biochem. Cell Biol..

[53] Li C.-Y., Pang Y.-Y., Yang H., Li J., Lu H.-X., Wang H.-L., Mo W.-J., Huang L.-S., Feng Z.-B., Chen G. (2017). Identification of miR-101-3p targets and functional features based on bioinformatics, meta-analysis and experimental verification in hepatocellular carcinoma. Am. J. Transl. Res..

[54] Liu Y., Tan J., Ou S., Chen J., Chen L. (2020). MicroRNA-101-3p suppresses proliferation and migration in hepatocellular carcinoma by targeting the HGF/c-Met pathway. Investig. New Drugs.

[55] Zhu W., Ni Q., Wang Z., Zhang R., Liu F., Chang H. (2025). MiR-101-3p targets the PI3K-AKT signaling pathway via Birc5 to inhibit invasion, proliferation, and epithelial–mesenchymal transition in hepatocellular carcinoma. Clin. Exp. Med..

[56] Hu S., Zhang J., Guo G., Zhang L., Dai J., Gao Y. (2022). Comprehensive analysis of GSEC/miR-101-3p/SNX16/PAPOLG axis in hepatocellular carcinoma. PLoS ONE.

