# Supplementary material for: Integrative Multi-Database Bioinformatics Characterization of FANCE in Hepatocellular Carcinoma: Prognostic, Epigenetic, Immune, and ncRNA Associations
Source: Medicina (Kaunas). 2026 Oct 14;62(10):1988. doi: 10.3390/medicina62101988 (PMC13706819; doi:10.3390/medicina62101988)
Supplement: Supplementary file 1 [file medicina-62-01988-s001.zip › Supplementary Materials.pdf]

Table S1. Prognostic value of *FANCE* expression across cancer cohorts.

Dataset	Cancer type	Endpoint	N	Cox *p*-value	HR (95% CI)
GSE9893	Breast cancer	Overall Survival	155	<0.001	6.66 (2.87–15.47)
MGH-glioma	Brain cancer	Overall Survival	50	<0.001	4.88 (2.06–11.53)
GSE13507	Bladder cancer	Disease-Specific Survival	165	<0.001	2.56 (1.50–4.38)
GSE11121	Breast cancer	Distant Metastasis-Free Survival	200	<0.01	2.71 (1.46–5.03)
GSE13507	Bladder cancer	Overall Survival	165	<0.01	1.71 (1.18–2.48)
jacob-00182-CANDF	Lung cancer	Overall Survival	82	<0.01	3.03 (1.39–6.61)
GSE13213	Lung cancer	Overall Survival	117	<0.01	1.93 (1.18–3.15)
GSE31210	Lung cancer	Recurrence-Free Survival	204	<0.05	2.66 (1.21–5.86)
GSE19234	Skin cancer	Overall Survival	38	<0.05	3.90 (1.28–11.87)
GSE12945	Colorectal cancer	Overall Survival	62	<0.05	5.67 (1.36–23.72)
GSE6532-GPL570	Breast cancer	Recurrence-Free Survival	87	<0.05	3.13 (1.11–8.83)
GSE6532-GPL570	Breast cancer	Distant Metastasis-Free Survival	87	<0.05	3.13 (1.11–8.83)
E-TABM-346	Blood cancer	Event-Free Survival	53	<0.05	0.45 (0.21–0.97)
GSE26712	Ovarian cancer	Disease-Free Survival	185	<0.05	0.64 (0.42–0.98)
HARVARD-LC	Lung cancer	Overall Survival	84	<0.05	0.27 (0.08–0.96)
GSE31210	Lung cancer	Overall Survival	204	<0.05	3.03 (1.03–8.91)
GSE3143	Breast cancer	Overall Survival	158	<0.05	0.55 (0.31–0.99)

* Cox *p*-valuse, HRs, and 95% Cis were obtained from the unvariable Cox regression results provided by Prognoscan

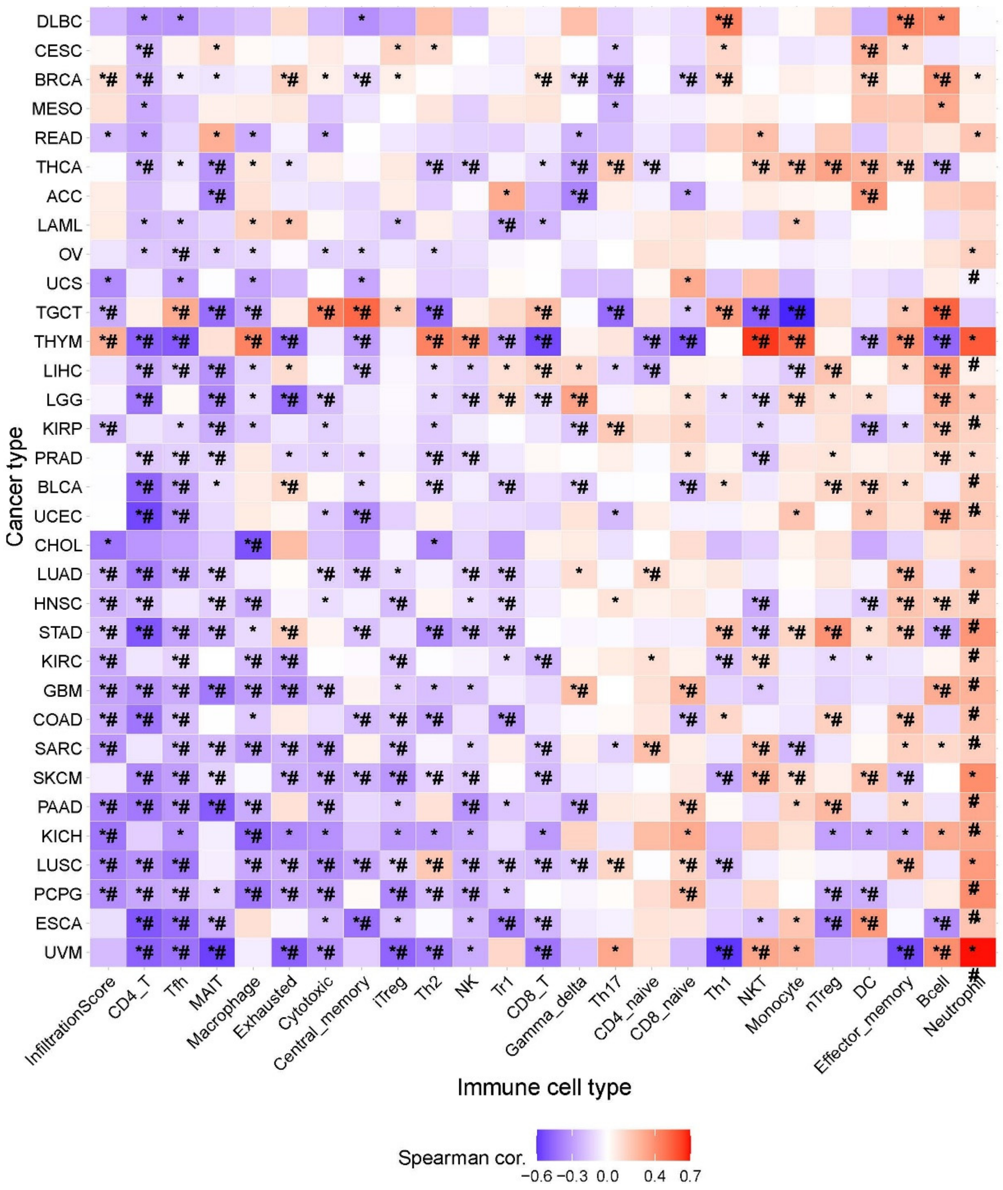

Figure S1. Association between *FANCE* expression and immune cell infiltration in various cancer types. Heatmap presenting the Spearman correlation coefficients for *FANCE* expression and the indicated immune cell populations. Color intensity reflects the direction and magnitude of the correlation. *p ≤ 0.05 and #FDR ≤ 0.05.

Abbreviations: ACC, adrenocortical carcinoma; BLCA, bladder urothelial carcinoma; BRCA, breast invasive carcinoma; CESC, cervical squamous cell carcinoma and endocervical adenocarcinoma; CHOL, cholangiocarcinoma; COAD, colon adenocarcinoma; DLBC, diffuse large B-cell lymphoma; ESCA, esophageal carcinoma; GBM, glioblastoma multiforme; HNSC, head and neck squamous cell carcinoma; KICH, kidney chromophobe; KIRC, kidney renal clear cell carcinoma; KIRP, kidney renal papillary cell carcinoma; LAML, acute myeloid leukemia; LGG, brain lower grade glioma; LIHC, liver hepatocellular carcinoma; LUAD, lung adenocarcinoma; LUSC, lung squamous cell carcinoma; MESO, mesothelioma; OV, ovarian serous cystadenocarcinoma; PAAD, pancreatic adenocarcinoma; PCPG, pheochromocytoma and paraganglioma; PRAD, prostate adenocarcinoma; READ, rectum adenocarcinoma; SARC, sarcoma; SKCM, skin cutaneous melanoma; STAD, stomach adenocarcinoma; TGCT, testicular germ cell tumors; THCA, thyroid carcinoma; THYM, thymoma; UCEC, uterine corpus endometrial carcinoma; UCS, uterine carcinosarcoma; UVM, uveal melanoma; CD4_T, CD4+

T cell; CD8_T, CD8+ T cell; DC, dendritic cell; MAIT, mucosal-associated invariant T cell; NK, natural killer cell; NKT, natural killer T cell; nTreg, natural regulatory T cell; Tfh, T follicular helper cell; Th1, T helper 1 cell; Th2, T helper 2 cell; Tr1, type 1 regulatory T cell; Treg, regulatory T cell
